# Multi-organ point-of-care ultrasound for COVID-19 (PoCUS4COVID): international expert consensus

**DOI:** 10.1186/s13054-020-03369-5

**Published:** 2020-12-24

**Authors:** Arif Hussain, Gabriele Via, Lawrence Melniker, Alberto Goffi, Guido Tavazzi, Luca Neri, Tomas Villen, Richard Hoppmann, Francesco Mojoli, Vicki Noble, Laurent Zieleskiewicz, Pablo Blanco, Irene W. Y. Ma, Mahathar Abd. Wahab, Abdulmohsen Alsaawi, Majid Al Salamah, Martin Balik, Diego Barca, Karim Bendjelid, Belaid Bouhemad, Pablo Bravo-Figueroa, Raoul Breitkreutz, Juan Calderon, Jim Connolly, Roberto Copetti, Francesco Corradi, Anthony J. Dean, André Denault, Deepak Govil, Carmela Graci, Young-Rock Ha, Laura Hurtado, Toru Kameda, Michael Lanspa, Christian B. Laursen, Francis Lee, Rachel Liu, Massimiliano Meineri, Miguel Montorfano, Peiman Nazerian, Bret P. Nelson, Aleksandar N. Neskovic, Ramon Nogue, Adi Osman, José Pazeli, Elmo Pereira-Junior, Tomislav Petrovic, Emanuele Pivetta, Jan Poelaert, Susanna Price, Gregor Prosen, Shalim Rodriguez, Philippe Rola, Colin Royse, Yale Tung Chen, Mike Wells, Adrian Wong, Wang Xiaoting, Wang Zhen, Yaseen Arabi

**Affiliations:** 1 Department of Cardiac Sciences, King Abdulaziz Medical City and King Abdullah International Medical Research Center, Riyadh, Saudi Arabia; 2 Cardiac Anesthesia and Intensive Care, Cardiocentro Ticino, Lugano, Switzerland; 3 New York Presbyterian Brooklyn Methodist Hospital, New York, NY USA; 4 Department of Medicine and Interdepartmental Division of Critical Care Medicine, University of Toronto, Toronto, Canada; 5 Department of Clinical-Surgical, Diagnostic and Paediatric Sciences, Unit of Anaesthesia and Intensive Care, University of Pavia, Pavia, Italy; 6 Anaesthesia and Intensive Care, Fondazione Istituto Di Ricovero E Cura a Carattere Scientifico, Policlinico San Matteo Foundation, Pavia, Italy; 7 Emergency Medicine and Critical Care Consultant, King Fahad Specialist Hospital – Dammam, Dammam, Saudi Arabia; 8 School of Medicine, Francisco de Vitoria University, Madrid, Spain; 9 University of South Carolina School of Medicine, Columbia, SC USA; 10 Anesthesia and Intensive Care, Fondazione IRCCS Policlinico San Matteo, Università Degli Studi Di Pavia, Pavia, Italy; 11 University Hospitals Cleveland Medical Center, Cleveland, OH USA; 12 Service D’Anesthésie Réanimation Hôpital Nord, APHM, Chemin des Bourrely, 13015 Marseille, France; 13 Department of Teaching and Research, Hospital “Dr. Emilio Ferreyra”, Necochea, Argentina; 14 Division of General Internal Medicine, Department of Medicine, University of Calgary, Calgary, Canada; 15 Emergency and Trauma Department, Hospital Kuala Lumpur, 50586 Kuala Lumpur, Malaysia; 16 King Abdulaziz Medical City, King Abdullah International Medical Research Center, Ministry of National Guard Health Affairs, Riyadh, Saudi Arabia; 17 College of Public Health and Health Informatics, King Saud Bin Abdulaziz University for Health Sciences, Riyadh, Saudi Arabia; 18 Dept of Anaesthesiology and Intensive Care, First Medical Faculty, Charles University, Prague, Czechia; 19 Médico Ecografista IADT, Buenos Aires, Argentina; 20 Intensive Care Division, Geneva University Hospitals, Geneva, Switzerland; 21 Department of Anaesthesiology and Intensive Care, C.H.U. Dijon and Université Bourgogne Franche-Comté, LNC UMR866, 21000 Dijon, France; 22 PICU Hospital San Juan de Dios, Santiago, Chile; 23 FOM University of Economy & Management, Frankfurt Campus, Frankfurt, Germany; 24 Hospital General, Instituto Mexicano del Seguro Social, De Zona 4 Monterrey, Nuevo Leon, Mexico; 25 Great North Trauma and Emergency Care Newcastle, Newcastle upon Tyne, UK; 26 Emergency Department, Latisana General Hospital, Latisana, Italy; 27 Department of Surgical, Medical and Molecular Pathology and Critical Care Medicine, University of Pisa, Pisa, Italy; 28 University of Pennsylvania, Philadelphia, PA USA; 29 Montreal Heart Institute, Montreal, Canada; 30 Medanta, The Medicity, Gurgaon, India; 31 Ospedale Niguarda C’ Grande, Milan, Italy; 32 Dept. of Emergency Medicine, Bundang Jesaeng Hospital, Seoul, Korea; 33 WINFOCUS Argentina BOD, Rosario, Argentina; 34 Department of Clinical Laboratory Medicine and Department of Emergency Medicine, Jichi Medical University, Tokyo, Japan; 35 Oregon Health and Science University, Portland, OR USA; 36 Department of Respiratory Medicine, Department of Clinical Research, Odense University Hospital, University of Southern Denmark, Odense, Denmark; 37 Khoo Teck Puat Hospital, Singapore, Singapore; 38 Dept. of Emergency Medicine, Yale School of Medicine, New Haven, CT USA; 39 Herzzentrum Leipzig, Leipzig, Germany; 40 Department of Ultrasound & Doppler Hospital de Emergencias “Dr. Clemente Alvarez”, Rosario, Santa Fe, Argentina; 41 Department of Emergency Medicine, Careggi University Hospital, Firenze, Italia; 42 Department of Emergency Medicine, Icahn School of Medicine At Mount Sinai, New York, NY USA; 43 Clinical Hospital Zemun, Faculty of Medicine, University of Belgrade, Belgrade, Serbia; 44 Faculty of Medecine, University of Lleida, Lleida, Spain; 45 Hospital Raja Permaisuri Bainun, Ipoh, Perak, Malaysia; 46 FAME - Medicine School of Barbacena - MG-Brasil, Barbacena, Brazil; 47 Arbo Education, Rio de Janeiro, Brazil; 48 SAMU 93 - Hôpital Avicenne, Paris, France; 49 Città Della Salute E Della Scienza Di Torino Hospital, University of Turin, Turin, Italy; 50 Faculty of Medicine and Pharmacy VUB, Univ Hospital Brussels, Brussels, Belgium; 51 Royal Brompton Hospital, London, UK; 52 Emergency Department, University Clinical Centre Maribor, Maribor, Slovenia; 53 Hospital Nacional Edgardo Rebagliati Martins, Lima, Peru; 54 Santa Cabrini Hospital, Montreal, Canada; 55 Department of Surgery, The University of Melbourne, Melbourne, VIC Australia; 56 Outcomes Research Consortium, Cleveland Clinic, Cleveland, OH USA; 57 Department of Emergency Medicine, Hospital Universitario La Paz, Madrid, Spain; 58 Division of Emergency Medicine, University of the Witwatersrand, Johannesburg, South Africa; 59 King’s College Hospital, London, UK; 60 Department of Critical Care Medicine, Peking Union Medical College Hospital, Chinese Academy of Medical Sciences & Peking Union Medical College, Beijing, 100730 China; 61 The Fourth Military Medical University, Xi’an, 710032 China; 62 King Abdulaziz Medical City, King Saud Bin Abdulaziz University for Health Sciences, King Abdullah International Medical Research Center, Riyadh, Saudi Arabia

**Keywords:** COVID-19, SARS-CoV-2, Point-of-care ultrasound (PoCUS), Focused cardiac ultrasound (FoCUS), Lung ultrasound (LUS), Echocardiography

## Abstract

COVID-19 has caused great devastation in the past year. Multi-organ point-of-care ultrasound (PoCUS) including lung ultrasound (LUS) and focused cardiac ultrasound (FoCUS) as a clinical adjunct has played a significant role in triaging, diagnosis and medical management of COVID-19 patients. The expert panel from 27 countries and 6 continents with considerable experience of direct application of PoCUS on COVID-19 patients presents evidence-based consensus using GRADE methodology for the quality of evidence and an expedited, modified-Delphi process for the strength of expert consensus. The use of ultrasound is suggested in many clinical situations related to respiratory, cardiovascular and thromboembolic aspects of COVID-19, comparing well with other imaging modalities. The limitations due to insufficient data are highlighted as opportunities for future research.
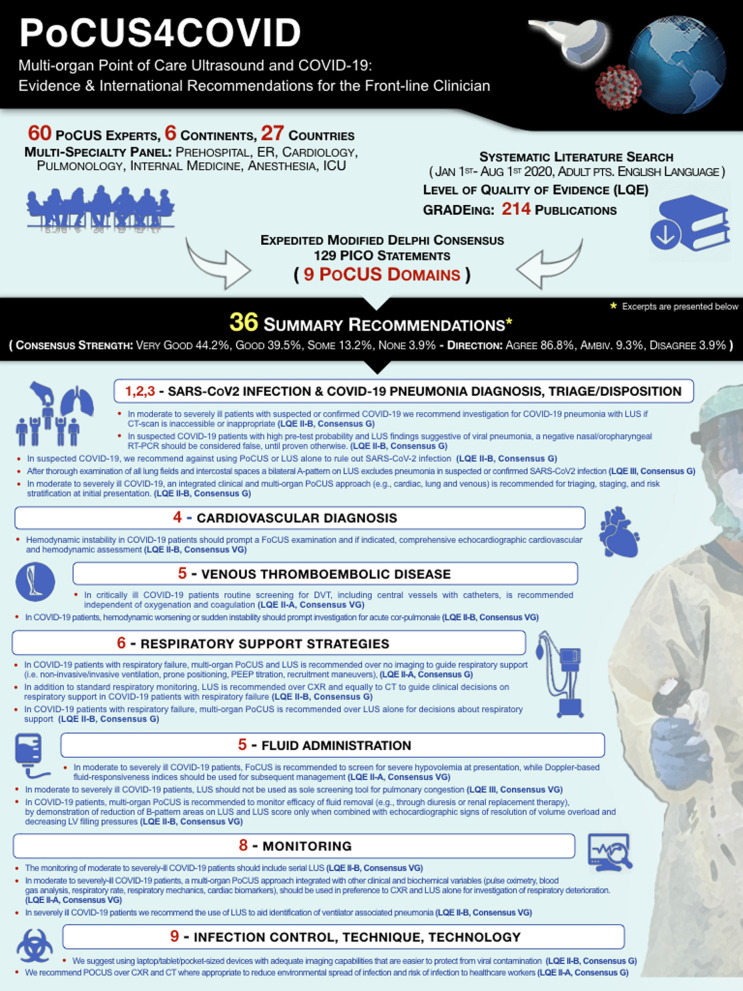

## Introduction

Since the first reports from China [1], SARS-CoV-2 has caused considerable morbidity and mortality from COVID-19 globally [1]. Although respiratory signs and symptoms are the most common manifestations, other systems may be involved [2]. Clinical presentations range from mild (80%) to life-threatening (5%), usually as acute respiratory distress syndrome (ARDS). Paucity of evidence, and urgency to adjust to evolving clinical scenarios have prompted adoption of approaches based on institutional experience [3], limited evidence, or extrapolation from other conditions [4, 5].

**Fig. 1:**
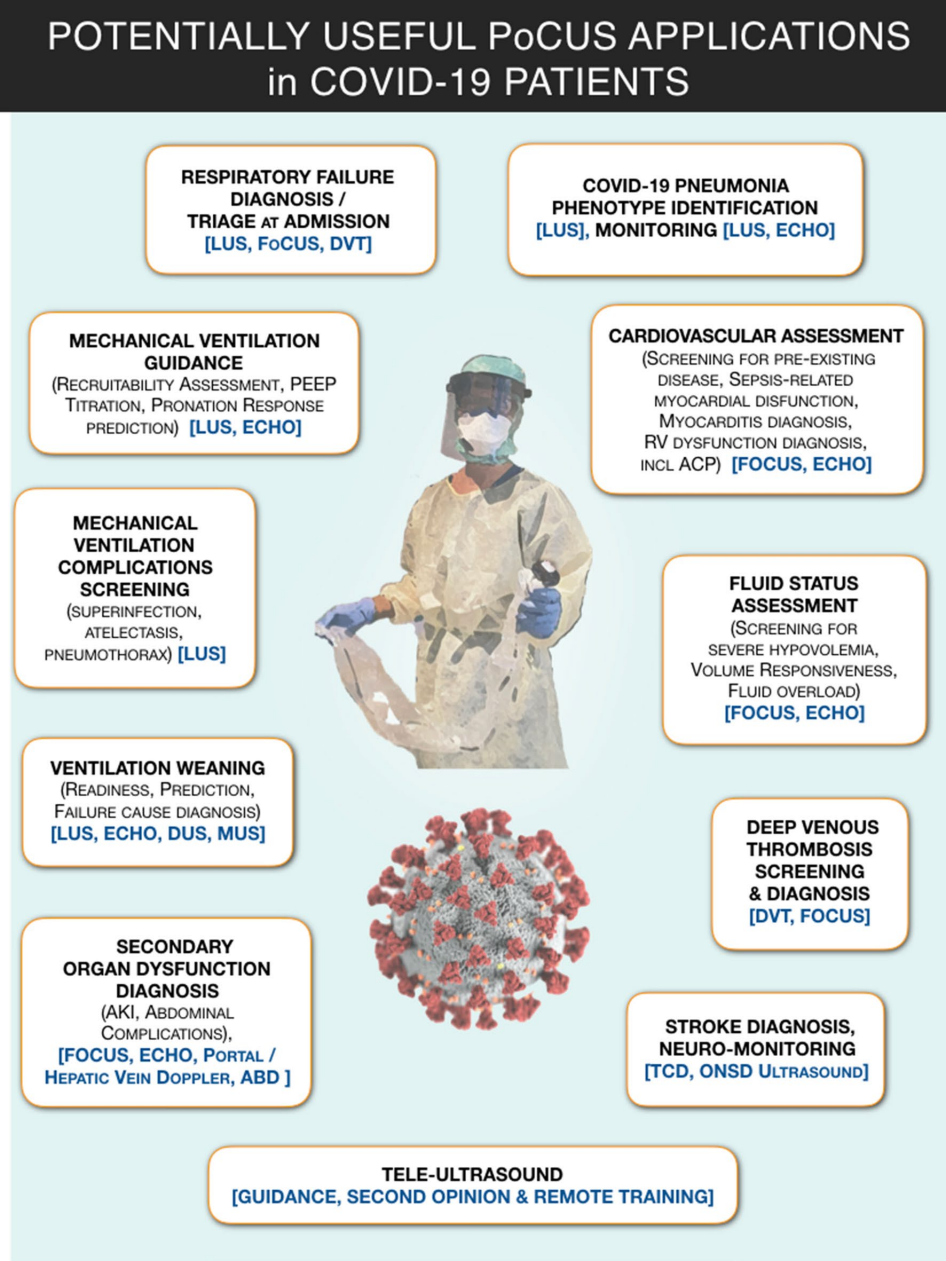
Graphical synopsis of potentially useful applications of point-of-care ultrasound (PoCUS) in COVID-19 patients. ABD, abdominal ultrasound; ACP, acute cor pulmonale; AKI, acute kidney injury; DUS, diaphragmatic ultrasound; DVT, ultrasound for deep venous thrombosis screening; ECHO, echocardiography; FoCUS, focused cardiac ultrasound; LUS, lung ultrasound; MUS, parasternal intercostal muscles ultrasound; ONSD, optic nerve sheath diameter; PEEP, positive end expiratory pressure; PoCUS, point-of-care ultrasound; TCD, transcranial Doppler; VASC, ultrasound for venous and arterial access

Point-of-care ultrasound (PoCUS) is a rapid, bedside, goal-oriented, diagnostic test that is used to answer specific clinical questions [6]. These distinctive features are appealing and address concerns of environmental contamination and disinfection of larger devices such as chest X-ray (CXR) and computed tomography (CT). Thus, multi-organ PoCUS could enhance the management of COVID-19 (Fig. 1).

## Methods

**Fig. 2:**
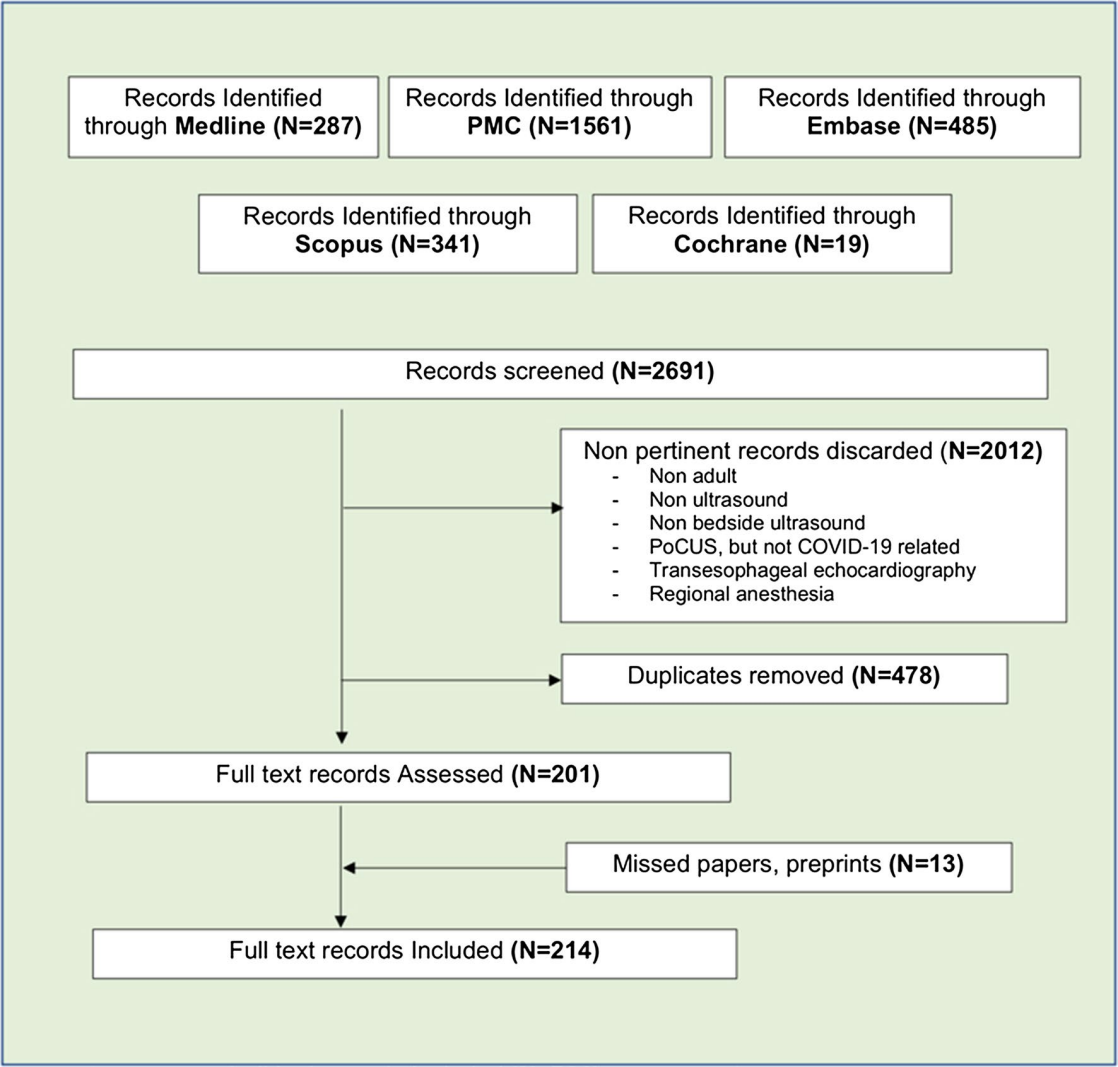
Literature search strategy. A literature search of Pubmed, Pubmed Central, Embase, Scopus and Cochrane library databases was conducted by 2 independent researchers from 01/01/2020–01/08/2020 to identify all publications on point-of-care ultrasound in COVID-19 adult patients, using English language restriction, and the following MeSH query: ((“lung” AND “ultrasound”) OR “echocardiography” OR “Focused cardiac ultrasound” OR “point-of-care ultrasound” OR “venous ultrasound”) AND (“COVID-19” OR “SARS-CoV2”). Non-pertinent findings were discarded. The references of relevant papers were hand-searched for missed papers. Duplicates were removed. An additional search of pre-print publications was made through ResearchGate, preprint online repositories and social medias

We searched Medline, Pubmed Central, Embase, Cochrane, Scopus and online pre-print databases from 01/01/2020 to 01/08/2020, and collected all English language publications on PoCUS in adult COVID-19 patients, using the MeSH query: [(“lung” AND “ultrasound”) OR “echocardiography” OR “Focused cardiac ultrasound” OR “point-of-care ultrasound” OR “venous ultrasound”] AND [“COVID-19” OR “SARS-CoV2”]. This systematic search strategy (Fig. 2) [Additional file 1A] identified 214 records.

The available evidence for PoCUS in COVID-19 was considered. Where such evidence was not available, non-COVID-19 data were used. We then applied an expedited 2-round modified Delphi process to elicit a consensus from an expert panel [Additional file 1A], who voted on PICO statements in 9 distinct domains (Table 1) ] [Additional file 1B] and approved the final recommendations. Consistent literature was GRADEd. Summary recommendations were generated based on voting results, literature evidence and experts’ input presented with Level of Quality of Evidence (LQE: I, II-A, II-B, III) and Level of Agreement (Very Good, Good, Some, None) [Additional file 1C] . Lastly, we identified limitations of PoCUS and areas of future research.

**Table 1:** PoCUS domains considered for consensus recommendations

Domain 1	PoCUS for Sars-Cov-2 infection diagnosis
Domain 2	PoCUS as a tool for triage/disposition
Domain 3	PoCUS for diagnosis of COVID-19 pneumonia
Domain 4	PoCUS for cardiovascular diagnosis
Domain 5	PoCUS for screening and diagnosis of thromboembolic disease
Domain 6	PoCUS and respiratory support strategies
Domain 7	PoCUS for management of fluid administration
Domain 8	PoCUS for monitoring of COVID-19 patients
Domain 9	PoCUS and infection control, techniques, technology and protocols

## DOMAINS 1—Diagnosis of SARS-CoV-2 infection, 2—Triage/disposition and 3—Diagnosis of COVID-19 pneumonia

COVID-19 almost invariably involves the respiratory system [2]. Approximately 5% of patients require critical care and mechanical ventilation, usually due to viral pneumonia and/or ARDS [7]. The diagnosis of COVID-19 pneumonia is challenging:

- Although CT has the best diagnostic yield [8], access is limited by patient volume, resources and risk of environmental contamination.
- Pre-existing conditions [9], and acute exacerbations of these diseases are common.
- Instability may preclude intra-hospital transportation.
- Delays or unreliability of reverse-transcriptase polymerase-chain-reaction (RT-PCR) results complicate infection control [10].
- Several algorithms/approaches developed for triage [11–20] are perceived as helpful, but remain unvalidated.

### Evidence

LUS is more accurate than CXR for diagnosing respiratory conditions [21], including interstitial diseases [22], pneumonia [23] and COVID-19 pneumonia [24]. The diagnostic accuracy of addition of LUS outperforms standard emergency department tests for dyspnea [25, 26]. LUS can diagnose COVID-19 pneumonia in patients with normal vital signs [27] and distinguish viral and bacterial pneumonias [28].

LUS findings associated with COVID-19 pneumonia are reported to be similar to previously described viral pneumonias [12, 22]. Frequently observed are [Additional files 2–5]: heterogeneous B-lines clusters, separated or confluent (corresponding to ground glass opacities on CT), large band-like longitudinal artifacts arising from normal pleural line (characterized as “light beam” [12]), pleural line irregularities, subpleural consolidations and areas with decreased lung sliding due to poor ventilation. Large consolidations with air bronchograms may be present, more commonly in patients requiring mechanical ventilation, possibly representing progression to ARDS or superimposed bacterial infection. At presentation, the distribution, although bilateral, is usually asymmetrical and patchy [29–31]. Lung involvement may be limited to dorsal/basal areas in milder COVID-19 pneumonia [32]. LUS shows good agreement with CT in recognizing lung pathology and its severity [33, 34] thus, identifying patients at higher risk of clinical deterioration, ICU admission, mechanical ventilation and mortality [34–36]. B-line count, consolidations and thickened pleural lines are associated with positive RT-PCR tests and clinical severity [37, 38]. Coupled with pretest probability, bilateral B-lines [single and/or confluent], irregular pleural line and subpleural consolidations increase the likelihood of diagnosing COVID-19 [39, 40], while non-specific, bilateral heterogeneous patterns [Additional file 6], combined with a typical clinical presentation, strongly suggest viral pneumonia. Conversely, if pre-test probability is low [41], a bilateral A-pattern on LUS may exclude COVID-19 pneumonia owing to its high negative predictive value for pneumonia [12, 30].

Multi-organ PoCUS yields a better diagnostic performance for causes of respiratory failure than LUS alone [42]. As a rapid, accurate diagnostic approach to acute dyspnea [43–45], it outperforms standard tests [26]. Similar results have been reported in undifferentiated shock [46]. PoCUS is recommended as a first-line diagnostic test for investigating respiratory failure and/or hypotension [22, 47]. PoCUS may raise suspicions of falsely negative RT-PCR and/or alternate diagnoses [48]. Recognition of comorbidities (chronic RV or LV dysfunction) and COVID-19-associated complications (DVT and RV failure) may influence patient disposition, and PoCUS can change their management [40].

We present a conceptual framework for triage of respiratory failure [Additional file 7]. Without more data, triage protocols cannot be developed that are universally applicable.

### Recommendations

1. We suggest using PoCUS, and especially LUS (presence of heterogeneous B-line clusters, pleural line irregularities, subpleural consolidations), and appropriately integrate the information with clinical assessment to diagnose COVID-19 pneumonia (LQE II-B, Very Good Agreement).
2. When CT-scan is not accessible or appropriate, we suggest using LUS to aid the diagnosis of COVID-19 pneumonia in suspected cases (LQE II-B, Good Agreement).
3. In patients with high pre-test probability for COVID-19 and LUS findings suggestive of pneumonia, a negative nasal/oropharyngeal RT-CR may not be used to exclude COVID-19, and LUS findings, further raising suspicion, should prompt repeat testing with better yield (LQE II-B, Good Agreement).
4. We do not recommend using PoCUS and LUS alone to rule out SARS-CoV-2 infection in suspected COVID-19 (LQE II-B, Good Agreement).
5. After thorough examination of all lung fields and intercostal spaces, a bilateral A-pattern suggests absence of pneumonia in suspected or confirmed SARS-CoV-2 infection (LQE III, Good Agreement).
6. We suggest multi-organ PoCUS integrated with other clinical information for triaging and risk stratification of suspected COVID-19 at initial presentation (LQE II-B, Good Agreement).

### Limitations and future research

More data are required to establish the accuracy of LUS findings for the diagnosis of COVID-19 pneumonia versus other viral pneumonias. PoCUS use for risk stratification, outcome prediction, and its impact on management of COVID-19 needs study.

## DOMAIN 4—Cardiovascular diagnosis in COVID-19

Numerous cardiovascular issues are associated with COVID-19:

- Patients with cardiovascular comorbidities seem to develop more severe COVID-19 [49].
- Up to 17% of hospitalized COVID-19 patients sustain acute cardiac injury (ACI) that increases mortality [50, 51–53]. Besides the inflammatory and direct cellular injury, other possible mechanisms for ACI include hypoxemia and result in oxygen supply/demand imbalance [54]. A close association of acute and fulminant myocarditis with COVID-19 is not established. However, if present, it will result in low output syndrome or cardio-circulatory collapse [55]. Though high-sensitivity troponin assays allow detection of myocardial injury, no cutoff values reliably distinguish myocardial infarction (MI) from other ACI [56]. Elevation of cardiac biomarkers, ECG changes, LV and RV dysfunction [57, 58] have been reported in myocarditis and AMI [55, 59].
- It is difficult to distinguish the effects of pneumonia from superimposed congestive heart failure [59].
- Respiratory acidosis, alveolar inflammatory edema and microvascular alterations may increase pulmonary vascular resistance [60], and positive pressure ventilation may further increase RV afterload, precipitating RV failure [61].
- Various cardiac manifestations [62] have been described, and some critically ill COVID-19 patients exhibit shock states [51].

### Evidence

Echocardiography and FoCUS are established tools for diagnosing cardiovascular disease [47, 63, 64]. FoCUS can detect pre-existing cardiac disease [Additional file 8] and acute RV and/or LV dysfunction [47]. Echocardiography [65] and FoCUS are recommended by American and European Echocardiography societies as diagnostic/monitoring tools in COVID-19 [66, 67]. FoCUS can guide decisions on coronary angiography [68] and inotropic/mechanical circulatory support [59, 69, 70]. Overt symptoms of myocardial ischemia, raised cardiac biomarkers, ECG changes and new LV regional wall motion abnormalities should be carefully evaluated so that myocardial infarction [Additional file 9] diagnostic/therapeutic pathways are followed expediently [54, 67, 68]. Low voltage QRS complexes, myocardial hyper-echogenicity, diffuse hypokinesia or regional wall motion abnormalities suggest myocarditis [71] [Additional file 11]. Acute cor-pulmonale can occur in COVID-19 [58, 72], and FoCUS can detect RV dilatation, paradoxical septal motion and RV longitudinal dysfunction [47] [Additional file 10]. Thus, FoCUS/echocardiography together with clinical and biochemical indices can enhance management of cardiovascular compromise.

### Recommendations

7. We suggest FoCUS and/or echocardiography assessment in moderate-severe COVID-19 as it may change clinical management or provide information that could be lifesaving (LQE II-B, Very Good Agreement).
8 We suggest FoCUS and/or echocardiography for assessment of hemodynamic instability in moderate-severe COVID-19 (LQE II-B, Very Good Agreement).
9 We recommend FoCUS and echocardiography to diagnose RV and LV systolic dysfunction and cardiac tamponade as etiology of hemodynamic instability in COVID-19 (LQE II-B, Very Good Agreement).
10 We suggest using FoCUS/echocardiography to guide hemodynamic management in severe COVID-19 (LQE II-B, Very Good Agreement).

### Limitations and future research

Whether subtypes of COVID-19 exist with more severe cardiovascular involvement and worse prognosis, requires investigation. Study of diastolic function may be of interest in COVID-19.

## DOMAIN 5—Screening and diagnosis of venous thromboembolic disease (VTE)

The risk of VTE in COVID-19 is high:

- Due to high incidence of DVT [73, 74] [Additional file 13].
- Pulmonary embolism (PE) [75, 76] [Additional file 10] and clotting in renal replacement circuits [75] in COVID-19 ICU patients are early and late complications.
- COVID-19 is associated with immunothrombotic dysregulation [77]. This manifests with high D-dimer [78], high C-reactive protein levels, anti-phospholipid antibodies [75] and sepsis-induced coagulopathy [79], and is likely to increase mortality [79].
- Screening for coagulopathy can risk stratify patients and may determine the need for anticoagulation [80]. However, higher D-dimer cutoffs may be needed to improve its specificity for DVT in COVID-19 [81].
- Whether DVT detection at hospital admission suggests more severe COVID-19 remains unknown.
- Despite standard thromboprophylaxis DVT is common in COVID-19 [81, 82].

### Evidence

Ultrasound is the mainstay of DVT diagnosis [83]. Screening is advised, when feasible, in the general management of COVID-19 patients [84]. Many factors limit access to formal duplex venous sonography [85]. Although routine screening is not widely recommended [86], twice weekly ultrasound surveillance can detect DVT, avert PE and reduce mortality in ICU patients [87].

Lower extremity ultrasound is recommended in COVID-19 patients with unexplained RV dysfunction, unexplained/refractory hypoxemia, or in patients with suspected PE who are too unstable for intra-hospital transport [86].

### Recommendations

11. Because critically ill COVID-19 patients have high risk for VTE, we suggest regular screening for DVT, including central vessels with catheters, independent of oxygenation and coagulation (LQE II-A, Very Good Agreement).
12 In moderate-severe COVID-19 with hemodynamic worsening or sudden instability, we suggest FoCUS for prompt investigation of acute cor-pulmonale (LQE II-B, Very Good Agreement).
13 In moderate-severe COVID-19, we suggest that echocardiographic indices of worsening RV function and/or increased pulmonary artery pressure may indicate PE (LQE II-A, Very Good Agreement).

### Limitations and future research

DVT prevalence and its role in risk stratification in mild COVID-19 are not known. Correlation of DVT with different COVID-pneumonia phenotypes needs study.

## DOMAIN 6—PoCUS and respiratory support strategies [including mechanical ventilation]

Phenotypes of COVID-19 pneumonia associated with similar degrees of hypoxemia but different lung weight, aerated volume and compliance have been described [88]. These range from “classic” ARDS (Phenotype-H) that responds to higher PEEP, to the better aerated low elastance (Phenotype-L) that often requires lower PEEP [89]. Future studies may clarify whether phenotyping COVID-19 pneumonia can guide respiratory support, mechanical ventilation settings, and minimize ventilator-induced lung injury [89].

“Classic” ARDS commonly involves dependent lung regions [90]; the same areas are typically involved in advanced COVID-19 pneumonia [89, 91]. Localizing consolidated lung is important to maximize benefit from prone positioning. Prone positioning is preferable when dorsal consolidation is severe with spared ventral zones [92]. Prone positioning in non-intubated patients may rapidly improve oxygenation [93, 94].

### Evidence

**Fig. 3:**
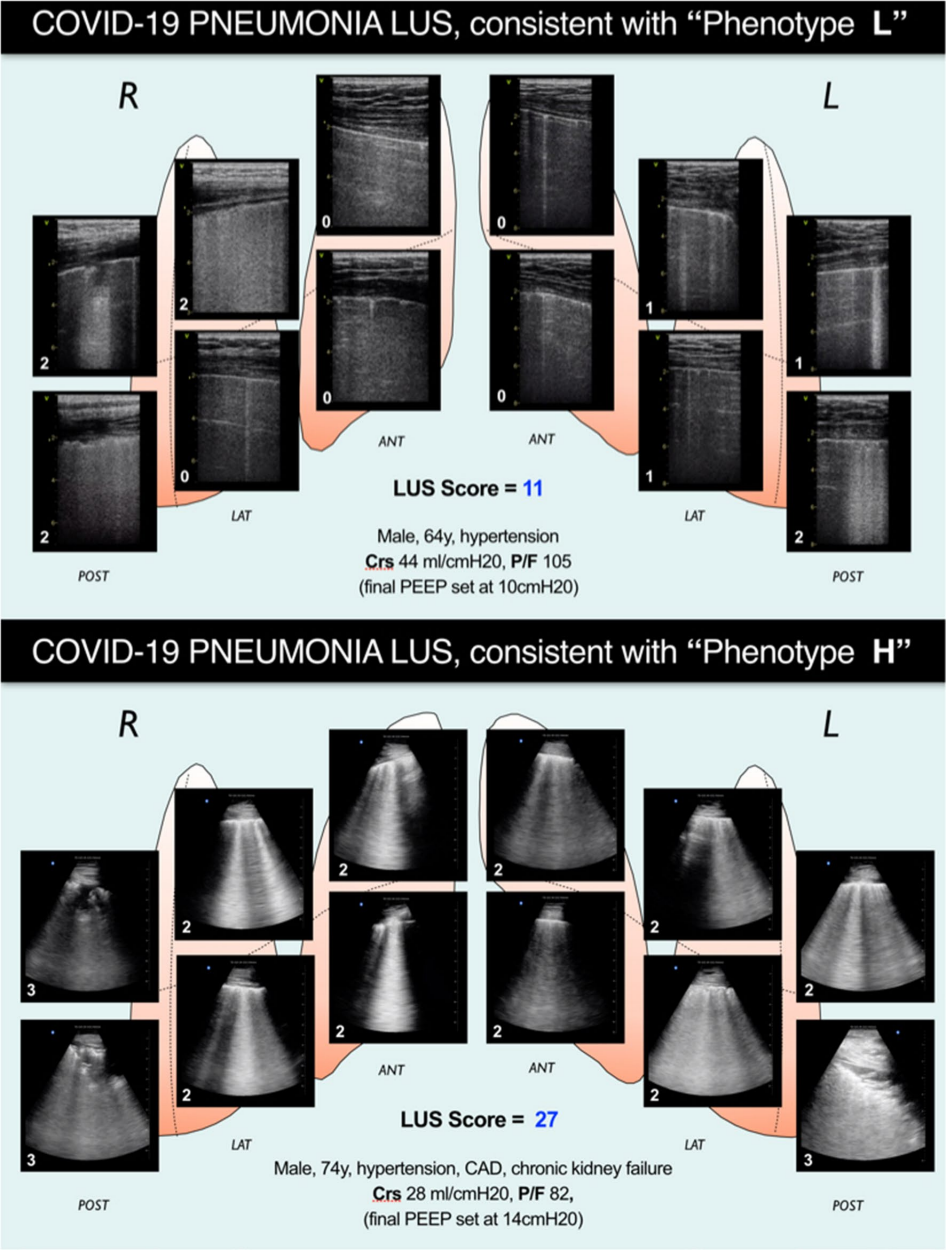
Examples of lung ultrasound cumulative patterns of patients presenting with a similar degree of hypoxemia, but very different degree of aeration and respiratory mechanics characteristics, and recalling the recently proposed COVID-19 pneumonia phenotypes [89]. Patient on upper panel presents a nearly normal respiratory system compliance and LUS evidence of a milder lung involvement, reflected in a total LUS score of 11. This suggests a lung condition matching which has been recently described as “Phenotype L,” based on CT findings, and characterized by low lung elastance and low ventilation/perfusion ratio (explaining the severe hypoxia). Based on this imaging and on respiratory mechanics findings, final PEEP was set at 10 cm H_2_0. Upper panel shows LUS evidence of a more diffuse and severe diffuse sonographic interstitial syndrome (cause of the shunt and the severe hypoxia), yielding a total LUS score of 27. Respiratory mechanics characteristics recall what has been described as “Phenotype H” (COVID-19 pneumonia: high lung elastance, high right-to-left shunt). Based on this imaging and on respiratory mechanics findings, PEEP was set at 14 cm H_2_0 after a stepwise recruiting maneuver. LUS, lung ultrasound

Like CT, LUS accurately characterizes regional lung pathology and identifies ARDS in COVID-19 pneumonia [33, 34, 40, 95]. LUS may discriminate mild-moderate from moderate-severe aeration loss, distinguishing different ARDS phenotypes [96] (Fig. 3).

Importantly, LUS may facilitate identification of patients with greater hypoxemia than expected for their alveolar lung injury (Fig. 3), in whom the pathophysiology may involve deranged perfusion (PE, micro-thrombosis, loss of pulmonary vasoconstriction, extrapulmonary shunt).

**Fig. 4:**
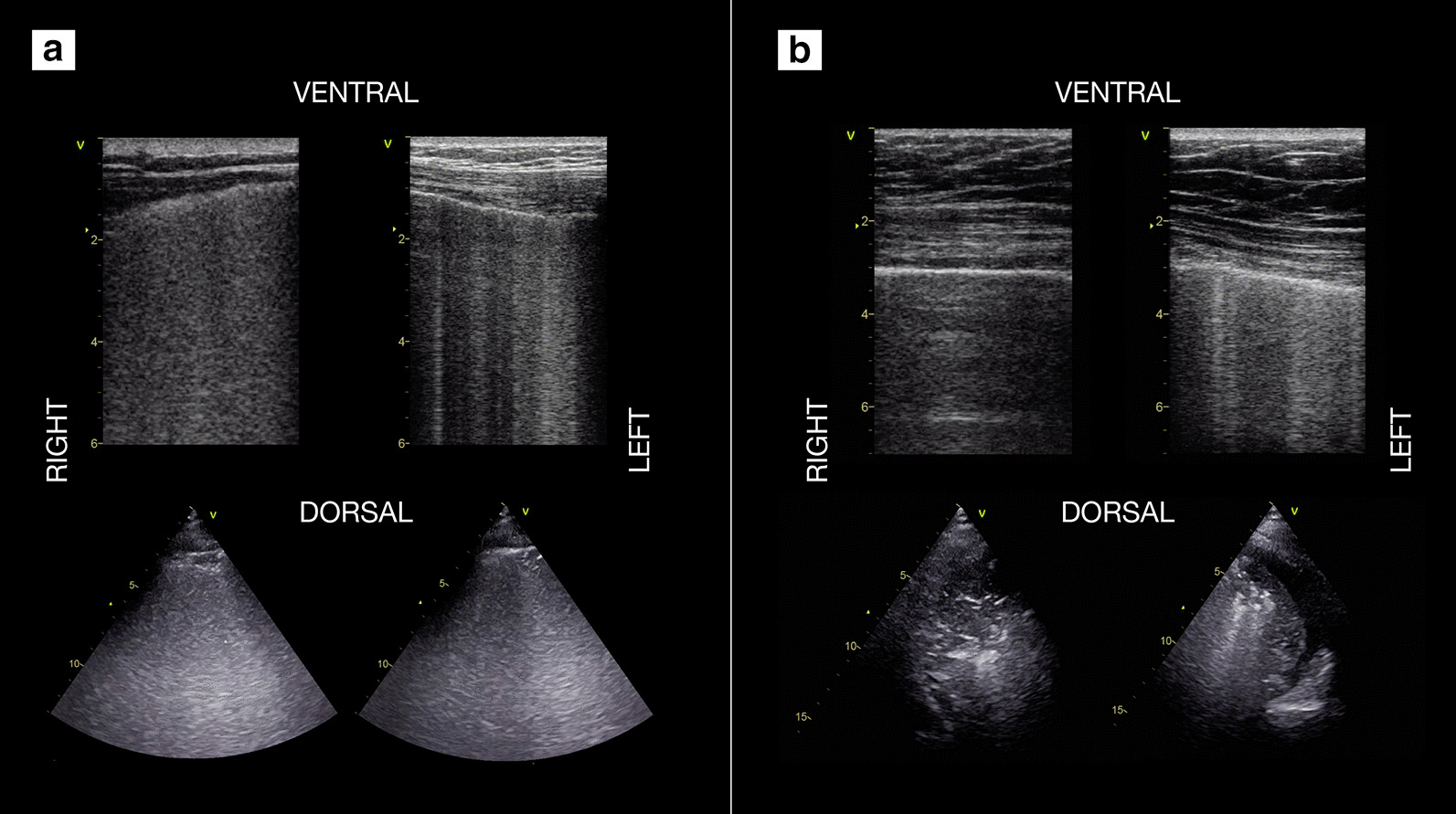
Use of lung ultrasound to monitor lung aeration and guide ventilatory management in 2 COVID-19 patients. **a** COVID-19 patient on day 2 after intubation and ICU admission, initially with PEEP 12 cmH_2_O: diffuse bilateral B-pattern with crowded, coalescent B-lines (“white lung appearance”) is visible, consistent with a sonographic interstitial syndrome and severe loss of aeration/increase of extravascular lung water. Based on these findings and on respiratory mechanics, a stepwise recruitment maneuver with a final PEEP set at 15 cmH_2_0 was performed, with improvement in gas exchange. **b** A different COVID-19 patient on day 4; PEEP set at 14 cmH_2_O: in comparison with previous patient, less B-lines are visible in ventral scans, with asymmetric distribution (more on the left scan); dorsal areas show lung consolidations, larger on the right side, with air bronchograms (dynamic at live scan). A pronation trial was successful, yielding immediate improvement in gas exchange and subsequent re-aeration of dorsal areas. (Ventral scans are taken with a linear, high frequency probe, dorsal ones with a phased array low-frequency one)

**Fig. 5:**
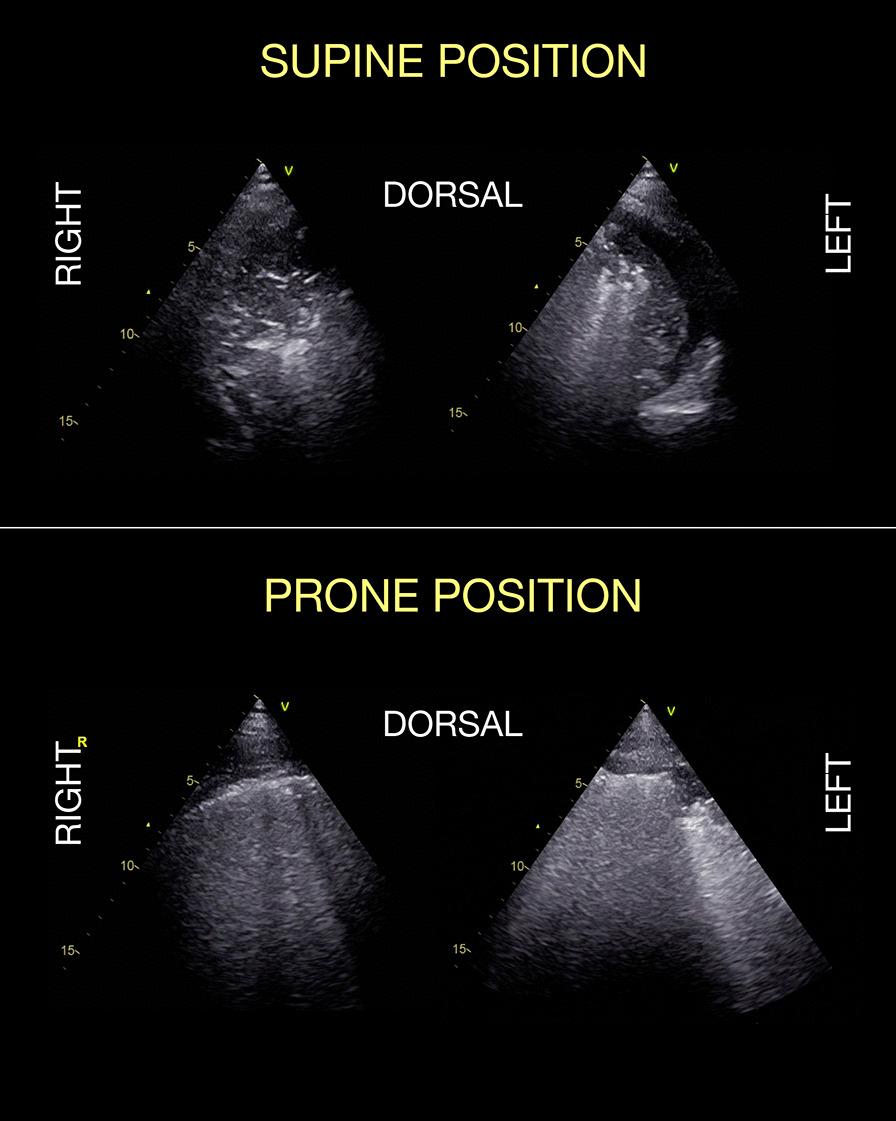
Lung ultrasound to monitor adequacy of re-aeration of dorsal areas upon pronation and recruitment maneuvers in a COVID-19 patient. Same patient of Fig. 2B, before (upper panels) and after (lower panels) pronation and a series of stepwise recruitment maneuvers up to PEEP 26 cmH_2_O, and final PEEP setting at 16 cmH_2_0

Global LUS score is strongly associated with lung tissue density/aeration measured with CT [97]. Using LUS to guide mechanical ventilation has been recommended [98] (Fig. 4). However, recruitment demonstrated by LUS correlates with recruitment estimated by pressure–volume curves [99], but not CT [97]. Although LUS may not predict oxygenation response to prone positioning, it does predict re-aeration of dorsal zones [100] (Fig. 5). LUS findings also correlate with extravascular lung water in ARDS [101, 102] and can monitor changes in aeration [103]. This has also been suggested in COVID-19 [104–106].

### Recommendations

14. We suggest multi-organ PoCUS including LUS over no imaging to guide respiratory support in COVID-19 with respiratory failure (i.e. ventilation, prone positioning, PEEP, recruitment maneuvers) (LQE II-A, Good Agreement).
15 In addition to standard respiratory monitoring, we suggest LUS over CXR and equally to CT, to guide clinical decisions on respiratory support in COVID-19 with respiratory failure (LQE II-B, Good Agreement).
16 We suggest multi-organ PoCUS over LUS alone for decisions about respiratory support in COVID-19 with respiratory failure (LQE II-B, Good Agreement).

### Limitations and future research

The benefit of LUS in ventilated COVID-19 patients is only theoretical. Studies to predict response to prone positioning, PEEP titration and other interventions are awaited. Role of LUS to decide invasive mechanical ventilation is unknown.

## DOMAIN 7—Management of fluid administration in COVID-19 patients

Fluid management is fundamentally important and often challenging in critically ill patients [107]. In COVID-19 patients, fluid overload can exacerbate lung dysfunction. Recent recommendations stress the need for conservative fluid strategies [4].

### Evidence

A large international survey found that PoCUS was the most frequently used approach to assess fluid responsiveness in critically ill COVID-19 patients [108]. While FoCUS can detect early signs of severe central hypovolemia [47] [Additional file 12], interpretation of inferior and superior vena cava collapsibility/distensibility indices is difficult when a variety of ventilation modalities are employed [18, 109]. Transesophageal echocardiography has inherent risks and limitations related to manpower and infection control [110].

Dynamic indices based on stroke volume variation, passive leg raising and mini-bolus administration techniques are good predictors of fluid responsiveness [111, 112] and can be assessed with transthoracic echocardiography.

In non-COVID-19 pneumonia patients, LUS has been shown to provide information on fluid tolerance and detect the consequences on the lung of overzealous fluid resuscitation [113, 114]. Resolution of B-lines during hemodialysis has been described [115] and also observed in COVID-19 patients [116, 117].

### Recommendations

17. We suggest FoCUS to screen for severe hypovolemia in moderate-severe COVID-19 at presentation, while Doppler-based fluid-responsiveness indices may be used for subsequent management (LQE II-A, Very Good Agreement).
18 We suggest that LUS alone is not sufficient as a screening tool for pulmonary congestion in moderate-severe COVID-19 (LQE III, Very Good Agreement).
19 We suggest that LUS alone is not sufficient to judge the appropriateness of fluid administration in moderate-severe COVID-19 (LQE II-B, Very Good Agreement).
20 In moderate-severe COVID-19, we suggest multi-organ PoCUS to monitor efficacy of fluid removal, by not only LUS findings of reduction of B-pattern areas, but also echocardiographic signs of resolution of volume overload and decreasing LV filling pressures (LQE II-B, Very Good Agreement).

### Limitations and future research

In COVID-19 pneumonia, the severity of the bilateral interstitial manifestations may either be due to variations in the inflammatory condition of the lung or changes due to pulmonary congestion. Simplified PoCUS-guided fluid management could be beneficial in resource-limited settings and needs further studies.

## DOMAIN 8—Monitoring patients with COVID-19

**PoCUS FOR RESPIRATORY MONITORING:** COVID-19 pneumonia is characterized by a wide spectrum of clinical presentations, from mild-moderate hypoxia to severe manifestations requiring life-sustaining measures [118]. In situations where large numbers of patients are admitted to areas with limited monitoring and staffing, disease progression may go unrecognized. Moreover, rapid progression to respiratory arrest has been reported [119]. Severe COVID-19 pneumonia is characterized by severe respiratory failure [120], but not necessarily as ARDS.

### Evidence

Evolution of LUS findings and their quantification using scoring systems are effective in monitoring progression or resolution of lung injury, especially in terms of variations in aeration and extravascular water content [22, 98, 103, 121, 122]. LUS is very sensitive, but is not specific enough to identify all causes of respiratory deterioration [22]. A comprehensive semi-quantitative LUS approach [97] can assess severity of lung injury and distribution patterns.

In patients with COVID-19 pneumonia, progression of LUS findings has been correlated with clinical and radiological deterioration. Thus, it can accurately monitor the evolution throughout its spectrum of severity, from mechanically ventilated [104, 105, 123] or veno-venous-ECMO patients [106], to milder cases [124,125, 126]. LUS has helped in identifying superimposed bacterial infections [127], and the response to antibiotic treatment [128]. LUS Monitoring has reduced use of CT and CXR in critically ill and COVID-19 populations [129, 130].

### Recommendations

21. We suggest serial LUS for respiratory monitoring in moderate-severe COVID-19 (LQE II-B, Very Good Agreement).
22 We suggest multi-organ PoCUS integrated with other clinical and biochemical variables, in preference to CXR for investigation of respiratory deterioration in moderate-severe COVID-19 (LQE II-A, Very Good Agreement).
23 We suggest multi-organ PoCUS over LUS alone to detect respiratory deterioration and guide treatment in moderate-severe COVID-19 (LQE II-B, Very Good Agreement).

### Limitations and future research

LUS has limitations and requires further research in early identification of patients who are more likely to progress to severe respiratory failure with inflammation, their pneumonia phenotype, and separate them from those with congestion.

**DETECTION OF MECHANICAL VENTILATION-RELATED COMPLICATIONS:** Approximately 2.5% of all COVID-19 patients [118] and up 88% of those admitted to ICU [9] require invasive mechanical ventilation, which may often last for weeks. The diagnosis of complications associated with prolonged ventilation requires imaging that may be limited due to risk of exposure to healthcare workers and environmental contamination. Thus, PoCUS, performed at the beside by the treating physician, may provide an accurate alternative.

### Evidence

*Pneumothorax.* LUS has significantly higher sensitivity than CXR for the diagnosis of pneumothorax [79% versus 40%], whereas specificity is equally excellent [131]. However, most of these data are from trauma and post-procedural studies and may overestimate diagnostic performance of LUS in COVID-19. The negative predictive value of LUS for pneumothorax is approximately 100% (if pleural sliding, lung pulse and B or C patterns are observed) [132].

*Ventilator-associated pneumonia.* In the appropriate context, large consolidations not responsive to recruitment maneuvers or suction [133] are highly suggestive of secondary bacterial infection [127, 134].

*Diaphragmatic dysfunction, and weaning failure from mechanical ventilation.* Ventilation-induced diaphragmatic injury can be reliably assessed with ultrasound [135]. Combining LUS score with the evaluation of LV and diaphragm function may improve the success of weaning trials [136–139]. Assessment of parasternal intercostal muscles thickening fraction seems promising for predicting weaning failure [140]. Detection and treatment of unresolved pulmonary conditions can facilitate weaning [141, 142].

*Acute cor-pulmonale.* The effects of mechanical ventilation on RV function have been well-described. Acute cor-pulmonale becomes an important factor to be considered in the ventilation strategy [61, 143].

### Recommendations

24. We suggest a prompt assessment of clinical deterioration with LUS for a timely and accurate bedside diagnosis of pneumothorax in severe COVID-19 (LQE II-B, Very Good Agreement).
25 We suggest LUS for early identification of ventilator-associated pneumonia in severe COVID-19 (LQE II-B, Very Good Agreement).
26 We suggest multi-organ PoCUS over CXR and CT to assess readiness for weaning, predict success and diagnose the cause(s) of weaning failure in COVID-19 (LQE II-B, Very Good Agreement).

### Limitations and future research

The safety and cost-saving impact of LUS in diagnosing complications of mechanical ventilation is yet to be demonstrated. A decision process based on PoCUS for tracheal extubation vs. tracheostomy mandates validation.

**PoCUS FOR HEMODYNAMIC MONITORING**

### Evidence

FoCUS and echocardiography are recommended for hemodynamic monitoring in critical care [47, 63, 64]. A recent survey found that ultrasound is the most frequently used monitoring tool to assess cardiac output and pulmonary artery pressures in critical COVID-19 patients [108].

### Recommendations

27. We suggest FoCUS and/or echocardiography for hemodynamic monitoring in moderate-severe COVID-19 (LQE II-A, Very Good Agreement).
28 We suggest integrating PoCUS-derived information with data from other devices used for hemodynamic monitoring in severe COVID-19 (LQE II-B, Very Good Agreement).

### Limitations and future research

Validated PoCUS-driven hemodynamic management protocols in COVID-19 are needed.

**PoCUS FOR** **MONITORING OF OTHER ORGANS:** Many critically ill COVID-19 patients develop secondary organ dysfunction, including acute kidney injury (AKI), liver injury, rhabdomyolysis and gastrointestinal complications [118, 144]. Hemodynamic factors and viral tropism for tubular cells may contribute to AKI [145]. Gastrointestinal complications may result from sepsis, deranged hemodynamics, or microvascular thrombosis [75]. Neurological complications are also not infrequent in COVID-19 [146].

### Evidence

PoCUS can exclude post- and pre-renal causes of AKI (by assessing volume status and hemodynamics). It can detect systemic and renal venous congestion, important factors in AKI [147, 148], acute gastrointestinal complications [149, 150] including cholestasis and bowel ischemia in COVID-19 patients [151]. The use of PoCUS for the diagnosis and management of neurological conditions is acknowledged [152] and may be applicable in COVID-19.

### Recommendations

29. We suggest PoCUS assessment for pre-renal causes of AKI, including hemodynamics and venous congestion in COVID-19 (LQE II-B, Very Good Agreement).

### Limitations and future research

Expertise and data on PoCUS applications to detect organ dysfunction in COVID-19 especially AKI and acute abdomen are limited and need further study.

## DOMAIN 9—Infection control, PoCUS technique, technology, and protocols

In the context of COVID-19:

- Interest in PoCUS has increased.
- Choice of machines is limited.
- Infection transmission to operators and environmental viral dissemination are serious concerns that may impact the quality of ultrasound examination and the choice of equipment.
- A systematic scanning approach is required to avoid missing or misinterpreting important findings.

### Evidence

**Fig. 6:**
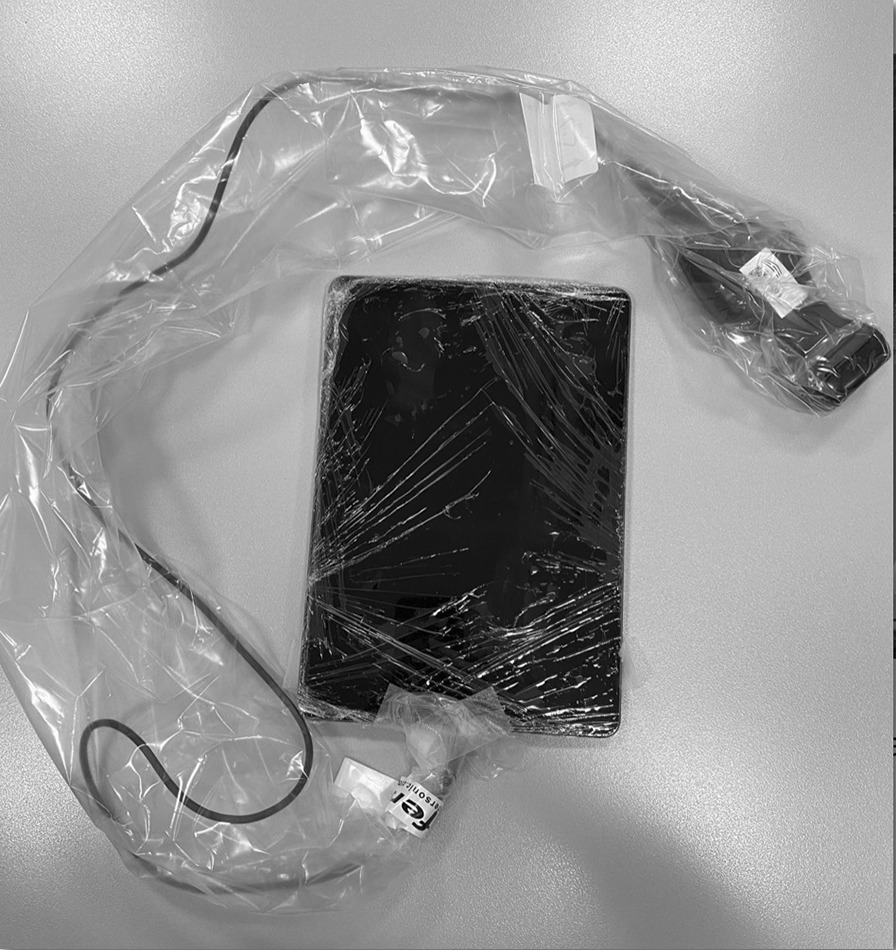
Example of highly portable ultrasound device covered for use on COVID-19 patients. The iPAD, with which the device works, is tightly enveloped in plastic film wrap, while the probe is covered with a dedicated sheath (normally used for sterile ultrasound-guided procedures). Donning and doffing the device requires assistance and involves stepwise uncovering, with multiple steps of disinfection before and after removing the covering. Use of the device is restricted to the COVID-19 unit

Laptop/tablet/pocket-sized machines provide reasonable compromise between portability and capability [153] (Fig. 6). Multi-frequency probes may be preferable to visualize both deep and superficial structures. While a single phased-array probe is suitable for FoCUS and LUS [154], a convex probe has been recommended by some experts [22]. Topographic zones and scanning techniques require standardization [12, 22, 30]. There is also a growing interest in telemedicine technology including robotic examinations [155] for remote guidance of minimally trained operators [156, 157] [Additional file 14].

To protect healthcare workers and patients, stringent infection control practices are crucial. Available guidance deals with environmental transmission and spread to personnel [158]. Recommendations on disinfectants [159] and information on SARS-CoV-2 survival on fomites [160] are also available.

### Recommendations

30. We suggest using laptop/tablet/pocket-sized devices with adequate imaging capabilities that are easier to protect from viral contamination in COVID-19 (LQE IIB, Good Agreement).
31. For diagnostic accuracy, quality control and obtaining second opinions, we suggest performance of standardized PoCUS examinations in COVID-19 (LQE II-B, Good Agreement).
32. We recommend reporting PoCUS studies and recording, storage and archiving of diagnostic images and cine-clips (LQE II-B, Good Agreement).
33. We suggest using tele-ultrasound for remote guidance and consultations in COVID-19. Simple audio-visual communication devices (e.g. smartphones) can facilitate this (LQE II-B, Good Agreement).
34. We suggest PoCUS over CXR and CT, where appropriate, to reduce environmental spread of infection and risk of infection to healthcare workers in COVID-19 (LQE IIA, Good Agreement).
35. We recommend strict adherence to manufacturers’ guidance for cleaning and disinfection of equipment used for COVID-19 (LQE II-A, Good Agreement).
36. We suggest brief and targeted ultrasound examinations to minimize cross-infection in COVID-19 (LQE II-B, Good Agreement).

### Limitations and future research

Information on quality, safety, remote mentoring/monitoring and archiving in COVID-19 is limited. Evidence for safety and efficacy of different disinfectants and methods of cleaning contaminated equipment is needed to make robust infection control policies.

## Conclusion

This consensus document based on the available evidence and expert opinion should encourage the use of PoCUS to improve patient outcomes during the current pandemic and development of meaningful protocols and practices to overcome COVID-19 and prepare for future challenges.

## Supplementary information

**Additonal file 1A.** Panel Composition, Literature search

**Additional file 1B.** Consensus Methodology

**Additional file 1C.** Consensus Results and Summary Recommendations

**Additional file 2.** (Video 1) Lung ultrasound (LUS) findings in COVID-19 Pneumonia. Clusters of B-lines. These usually have a patchy distribution

**Additional file 3.** (Video 2) Lung ultrasound (LUS) findings in COVID-19 Pneumonia. Longitudinal bright, band-like, large artifacts

**Additional file 4.** (Video 3) Lung ultrasound findings (LUS) in COVID-19 Pneumonia. Subpleural consolidations and spared areas

**Additional file 5.** (Video 4) Lung ultrasound (LUS) findings in COVID-19 Pneumonia. Lung consolidations in dorsal areas

**Additional file 6.** (Video 5). Cumulative lung ultrasound pattern in a patient with COVID-19 pneumonia. The exam was performed considering 3 regions per hemithorax (anterior, lateral and a posterior, with the sternum, the anterior axillary line and the posterior axillary line as landmarks) and an upper and a lower quadrant for each one of them. The resulting 6 areas per hemithorax are labelled with numbers from 1 to 6, and with L for left side and R for the right side

**Additional file 7.** PoCUS-empowered triage in respiratory failure during COVID-19 Pandemic. Conceptual framework of point-of-care ultrasound (PoCUS) use for the triage of dyspneic and/or hypoxemic patients, during the SARS-CoV-2 pandemic: the diagram does not represent an algorithm but rather a framework for potentially developing protocols according to local/institutional clinical practices, policies and regulations. It does not either provide a list of conclusive diagnosis or specific treatments, but suggests how to integrate at best PoCUS in the workflow of this specific setting

**Additional file 8.** (Video 6). Focused cardiac ultrasound (FoCUS) findings in a patient with COVID-19 Pneumonia and pre-existing cardiac disease. First panel shows a videoclip with findings consistent with chronic right ventricular dysfunction. Second panel shows videoclips with evidence of chronic left ventricular failure

**Additional file 9.** (Video 7). Focused cardiac ultrasound (FoCUS) findings in a patient with COVID-19 pneumonia and acute myocardial infarction.

**Additional file 10.** (Video 8). Focused Cardiac Ultrasound (FoCUS) findings in a patient with COVID-19 Pneumonia and acute cor pulmonale, due to both mechanical ventilation and submassive pulmonary embolism.

**Additional file 11.** (Video 9). Focused cardiac ultrasound (FoCUS) findings in a patient with COVID-19 pneumonia and myocarditis.

**Additional file 12.** (Video 10) Focused cardiac ultrasound (FoCUS) findings in a patient with COVID-19 pneumonia and severe hypovolemia.

**Additional file 13.** (Video 11). Focused cardiac ultrasound (FoCUS) findings in a patient with COVID-19 pneumonia and diffuse deep venous thrombosis. (Courtesy of Dr. Scopigni Francesca)

**Additional file 14.** (Video 12). Remote guidance with tele-ultrasound in the COVID-ICU. Operators within the isolation room perform lung and cardiac ultrasound exam in a COVID-19 pneumonia patient, with guidance and second opinion from a colleague in the non-COVID zone of the hospital. Guidance is provided verbally and with remote control of the ultrasound settings. (Courtesy of Dr. Bruno Capelli)

## Data Availability

The data and other material can be made available to the Journal.

## Abbreviations

ACI: Acute cardiac injury
ARDS: Acute respiratory distress syndrome
AKI: Acute kidney injury
COVID-19: Corona virus disease 19
CT: Computerized tomography
CXR: Chest radiography
DVT: Deep venous thrombosis
ECG: Electro-cardiogram
FoCUS: Focused cardiac ultrasound
ICU: Intensive care unit
LQE: Level of quality of evidence
LUS: Lung ultrasound
LV: Left ventricle
PE: Pulmonary embolism
PEEP: Positive end expiratory pressure
PICO: Patient intervention comparator outcome
PLR: Passive leg raising
PoCUS: Point-of-care ultrasound
RT-PCR: Reverse-transcriptase polymerase-chain-reaction
RV: Right ventricle
SARS-CoV-2: Severe acute respiratory syndrome corona virus-2
SVV: Stroke volume variation
TAPSE: Tricuspid annular plane systolic excursion
VTE: Venous thromboembolic disease
