# Supplementary material for: Multi-organ point-of-care ultrasound for COVID-19 (PoCUS4COVID): international expert consensus
Source: Crit Care. 2020 Dec 24;24:702. doi: 10.1186/s13054-020-03369-5 (PMC7759024; doi:10.1186/s13054-020-03369-5)
Supplement: Supplementary file 1 — Additonal file 1A. Panel Composition, Literature search [file 13054_2020_3369_MOESM1_ESM.pdf]

PoCUS4COVID

Expedited Delphi consensus process on the use of
Point of Care UltraSound for COVID Diagnosis and Management

N	Family Name	Name	Gender	Country	Specialty
1	HUSSAIN	Arif	M	KSA	INTENSIVE CARE - ANESTHESIA
2	ALSAAWI	Abdulmohsen	M	KSA	EMERGENCY MEDICINE
3	SALAMAH	Majid	M	KSA	EMERGENCY MEDICINE
4	NERI	Luca	M	KSA	EMERGENCY MEDICINE
5	ARABI	Yaseen	M	KSA	INTENSIVE CARE
6	GRACI	Carmela	F	ITALY	PREHOSPITAL EMERGENCY MEDICINE
7	CORRADI	Francesco	M	ITALY	EMERGENCY MEDICINE
8	PIVETTA	Emanuele	M	ITALY	EMERGENCY MEDICINE
9	COPETTI	Roberto	M	ITALY	EMERGENCY MEDICINE
10	NAZERIAN	Peiman	M	ITALY	EMERGENCY MEDICINE
11	TAVAZZI	Guido	M	ITALY	INTENSIVE CARE - ANESTHESIA
12	MOJOLI	Francesco	M	ITALY	INTENSIVE CARE - ANESTHESIA
13	BENDJELID	Karim	M	SWITZERLAND	INTENSIVE CARE - ANESTHESIA
14	VIA	Gabriele	M	SWITZERLAND	INTENSIVE CARE - ANESTHESIA
15	NESKOVIC	Aleksandar N	M	SERBIA	CARDIOLOGY
16	PROSEN	Gregor	M	SLOVENIA	PREHOSPITAL EMERGENCY MEDICINE
17	BALIK	Martin	M	CZECK REPUBLIC	INTENSIVE CARE - ANESTHESIA
18	POELAERT	Jan	M	BELGIUM	INTENSIVE CARE - ANESTHESIA
19	MEINERI	Massimiliano	M	GERMANY	INTENSIVE CARE - ANESTHESIA
20	BREITKREUTZ	Roul	M	GERMANY	EMERGENCY MEDICINE
21	LAURSEN	Christian	M	DENMARK	PULMONOLOGY
22	BOUHEMAD	Belaid	M	FRANCE	INTENSIVE CARE - ANESTHESIA
23	PETROVIC	Tomislav	M	FRANCE	PREHOSPITAL EMERGENCY MEDICINE
24	ZIELESKIEWICZ	Laurent	M	FRANCE	INTENSIVE CARE
25	PRICE	Susanna	F	UK	INTENSIVE CARE - CARDIOLOGY
26	WONG	Adrian	M	UK	INTENSIVE CARE - ANESTHESIA
27	CONNOLLY	Jim	M	UK	EMERGENCY MEDICINE
28	NOGUE	Ramon	M	SPAIN	EMERGENCY MEDICINE
29	TUNG-CHEN	Yale	M	SPAIN	EMERGENCY MEDICINE
30	VILLEN	Tomas	M	SPAIN	EMERGENCY MEDICINE
31	WELLS	Mike	M	SOUTH AFRICA	EMERGENCY MEDICINE
32	GOFFI	Alberto	M	CANADA	INTENSIVE CARE
33	ROLA	Philippe	M	CANADA	EMERGENCY MEDICINE
34	MA	Irene	F	CANADA	INTERNAL MEDICINE
35	DENAULT	André	M	CANADA	INTENSIVE CARE - ANESTHESIA
36	HOPPMANN	Richard	M	USA	INTERNAL MEDICINE
37	NOBLE	Vicki	F	USA	EMERGENCY MEDICINE
38	DEAN	Anthony	M	USA	EMERGENCY MEDICINE
39	MELNIKER	Larry	M	USA	EMERGENCY MEDICINE
40	LANSPA	Micheal	M	USA	INTENSIVE CARE
41	LIU	Rachel	F	USA	EMERGENCY MEDICINE
42	NELSON	Brett	M	USA	EMERGENCY MEDICINE
43	PEREIRA	Elmo	M	BRASIL	INTENSIVE CARE
44	PAZELI	José	M	BRASIL	INTERNAL MEDICINE
45	CALDERON	Juan	M	MEXICO	INTENSIVE CARE
46	HURTADO	Laura	F	ARGENTINA	INTERNAL MEDICINE
47	MONTORFANO	Miguel	M	ARGENTINA	EMERGENCY MEDICINE
48	BLANCO	Pablo	M	ARGENTINA	INTENSIVE CARE
49	BARCA	Diego	M	ARGENTINA	EMERGENCY MEDICINE
50	RODRIGUEZ	Shalim	M	PERÚ	EMERGENCY MEDICINE
51	BRAVO-FIGUEROA	Pablo	M	CHILE	INTENSIVE CARE
52	KAMEDA	Toru	M	JAPAN	INTENSIVE CARE
53	LEE	Frances	M	SINGAPORE	EMERGENCY MEDICINE
54	OSMAN	Adi	M	MALAYSIA	EMERGENCY MEDICINE
55	ABDULWAHAB	Mahatar	M	MALAYSIA	EMERGENCY MEDICINE
56	XIAOTING	Wang	M	CHINA	INTENSIVE CARE
57	ZHEN	Wang	M	CHINA	INTERNAL MEDICINE
58	HA	Young-Rock	M	SOUTH KOREA	EMERGENCY MEDICINE
59	GOVIL	Deepak	M	INDIA	INTENSIVE CARE
60	ROYSE	Colin	M	AUSTRALIA	ANESTHESIA

PoCUS4COVID

Expedite Delphi consensus process on the use of
Point of Care UltraSound for COVID Diagnosis and Management

LITERATURE SEARCH

A literature search of Pubmed, Pubmed Central, Embase, Scopus and Cochrane library databases was conducted by 2 independent researchers for the period Jan 1st 2020-August 1st 2020 to identify all publications on point of care ultrasound in COVID-19 adult patients, using English language restriction, and the following MeSH query: ((“lung” AND “ultrasound”) OR “echocardiography” OR “Focused cardiac ultrasound” OR “point of care ultrasound” OR “venous ultrasound”) AND (“COVID-19” OR “SARS-CoV2”). Non-pertinent findings were discarded. The references of relevant papers were also scanned for any missed paper. Duplicates were removed. An additional search of pre-print publications was made through ResearchGate, preprint online repositories and social medias.

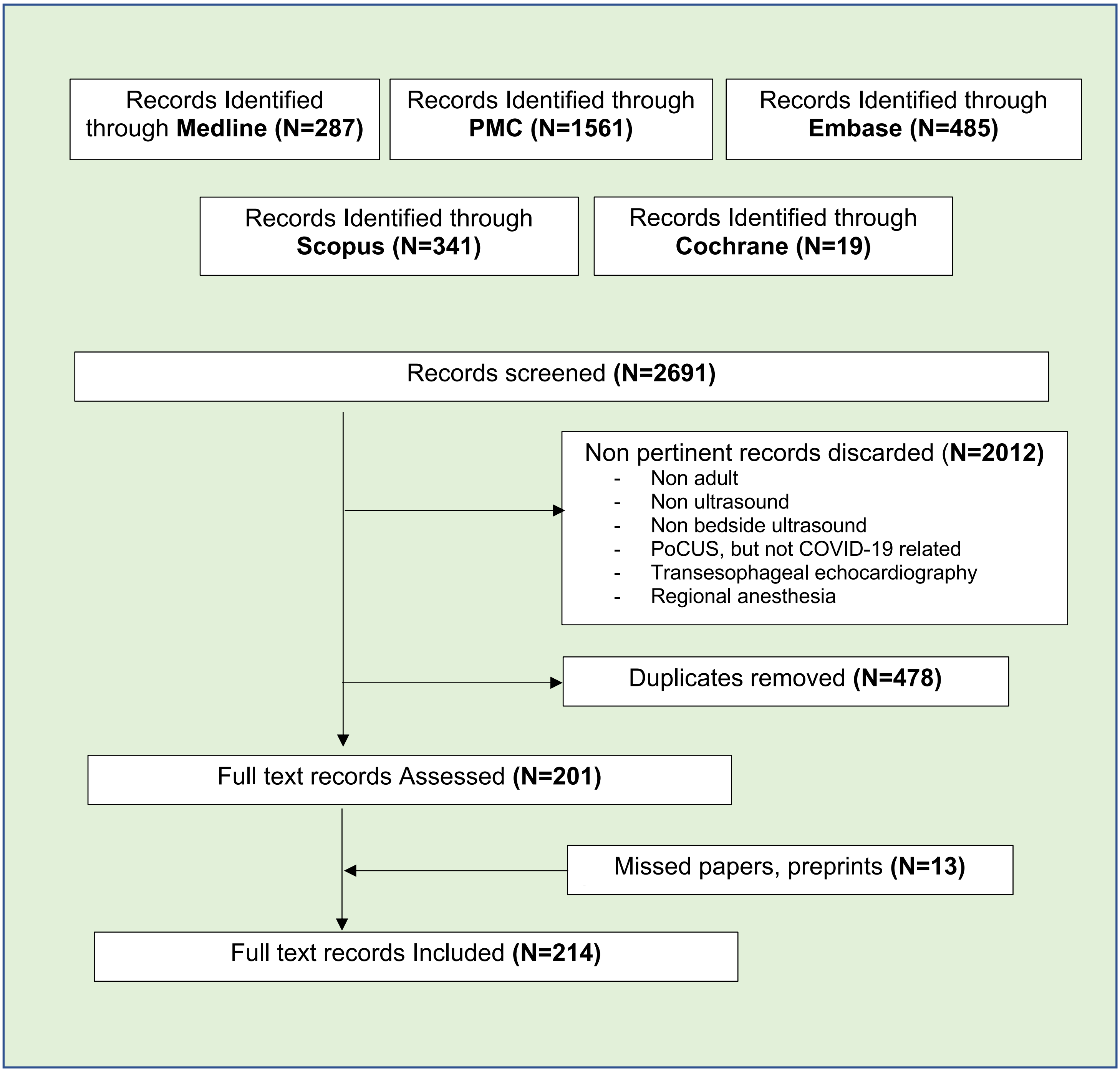

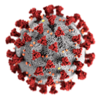

PoCUS4COVID

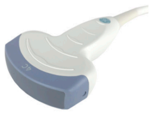

Expedited Delphi consensus process on the use of
Point of Care UltraSound for COVID Diagnosis and Management

1	Abramowicz JA.	WOFUMB POSITION STATEMENT HOW TO PERFORM A SAFE ULTRASOUND EXAMINATION AND CLEAN EQUIPMENT IN THE CONTEXT OF COVID-19	ULTRASOUND MED BIOL	2020
2	Abrams E.	Lung Ultrasound in COVID-19	ACEP	2020
3	Abrams ER.	POINT-OF-CARE ULTRASOUND IN THE EVALUATION OF COVID-19	J EMERG MED	2020
4	Akl EA	Use of Chest Imaging in the Diagnosis and Management of COVID-19/ a WHO Rapid Advice Guide	RADIOLOGY	2020
5	Alkafaji M.	Visual Case Discussion. A Case of Lung Ultrasound Findings in a 73-year-old male with COVID-19	VIS J EMERG MED	2020
6	Allinovi M.	Lung ultrasound may support diagnosis and monitoring of COVID-19 pneumonia	ULTRASOUND MED BIOL	2020
7	Anile A	COVID/ the new ultrasound alphabet in SARS-CoV-2 era	ANESTH ANALG	2020
8	Antunez-Montes OY.	RATIONALE FOR THE ROUTINE APPLICATION OF LUNG ULTRASOUND IN THE MANAGEMENT OF CORONAVIRUS DISEASE 2019 (COVID-19) PATIENTS IN MIDDLE- TO LOW-INCOME COUNTRIES	ULTRASOUND MED BIOL	2020
9	Antunez-Montes OY.	Routine use of Point-of-Care lung ultrasound during the COVID-19 pandemic	MED INTENSIVA	2020
10	Artifoni M.	Systematic assessment of venous thromboembolism in COVID-19 patients receiving thromboprophylaxis/ incidence and role of D-dimer as predictive factors	J THROMB THROMBOL	2020
11	Avinash A.	Lung Ultrasound in COVID-19	AFR J EM MED	2020
12	Baker K.	Lung ultrasound in a COVID pandemic - choosing wisely	AUSTRALAS J ULTRASOUND MED	2020
13	Bar S.	Association of lung ultrasound images with COVID-19 infection in an emergency room cohort	ANAESTH	2020
14	Benchoufi M.	Lung injury in patients with or suspected COVID-19 / a comparison between lung ultrasound and chest CT-scanner severity assessments, an observational study	medXRiv	2020
15	Bhandari S.	Evaluation of Clinico–Radiological Profile and Correlation with Ultrasonography of the Chest in Coronavirus Disease 2019 Pneumonia	INDIAN J MED SPEC	2020
16	Bhayana R.	Abdominal Imaging Findings in COVID-19/ Preliminary Observations	RADIOLOGY	2020
17	Bhimji-Hewitt S.	Can the Use of Bedside Lung Ultrasound Reduce Transmission Rates in The Case of The COVID-19 Patient? - A Narrative Review	POCUS	2020
18	Bhoi S.	Point-of-care ultrasound in COVID-19 pandemic	POSTGRAD MED J	2020
19	Boero E.	The role of lung ultrasonography in COVID-19 disease management	JACEP	2020
20	Bonadia N.	Lung Ultrasound findings are associated with mortality and need of intensive care admission in COVID-19 patients evaluated in the Emergency Department	ULTRASOUND MED BIOL	2020
21	Born J.	POCOVID-Net/ AUTOMATIC DETECTION OF COVID-19 FROM A NEW LUNG ULTRASOUND IMAGING DATASET (POCUS) arXiv	arXiv	2020
22	Buda N.	Consensus of the Study Group for Point-of-Care Lung Ultrasound in the intensive care management of COVID-19 patients	ANAESTHESIOL INTENSIVE THER	2020
23	Buda N.	Lung ultrasound in the diagnosis of COVID-19 infection - A case series and review of the literature	ADVANC MED SCI	2020
24	Buonsenso D.	Clinical role of lung ultrasound for the diagnosis and monitoring of COVID-19 pneumonia in pregnant women	ULTRASOUND OBSTET GYNECOL	2020
25	Buonsenso D.	COVID-19 outbreak; less stethoscope, more ultrasound	LANCET RESP	2020
26	Buonsenso D.	Effectiveness of a ‘fast lung ultrasound teaching program’ for gynecologists/obstetricians dealing with pregnant women with suspicion of COVID-19 infection	ULTRASOUND OBSTET GYNECOL	2020
27	Buonsenso D.	Point-of-Care Lung Ultrasound findings in novel coronavirus disease-19 pnemoniae; a case report and potential applications during COVID-19 outbreak	EUR REV MED PHARMACOL SCI	2020
28	Cameli M.	Safe performance of echocardiography during the COVID-19 pandemic_ a practical guide	REV CARDIOVASC RES	2020
29	Chen L.	Novel Coronavirus-Induced Right Ventricular Failure and Point of Care Echocardiography; A Case Report	CARDIOLOGY	2020
30	Chliem A.	Pick Up Your Probes A Call for Clinically Oriented Point-of-Care Ultrasound Research in COVID-19	J ULTRASOUND MED	2020
31	Cho YJ.	Lung ultrasound for early diagnosis and severity assessment of pneumonia in patients with coronavirus disease 2019	KOREAN J INTERN MED	2020
32	Chun-Hei Cheung J.	POCUS in COVID-19/ pearls and pitfalls	LANCET RESP MED	2020
33	Churchill TW.	Echocardiographic Features of COVID-19 Illness and Association with Cardiac Biomarkers	JASE	2020
34	Consoli L.	2019 novel coronavirus (COVID-19) pneumonia complications/ the importance of lung ultrasound	J ULTRASOUND	2020
35	Convissar D.	Application of Lung Ultrasound During the Coronavirus Disease 2019 Pandemic; A Narrative Review	ANESTHA ANALG	2020
36	Copetti R.	First comes the A, then the B; what we learned from the COVID-19 outbreak	EUR J INTERN MED	2020
37	Cosyns B.	The role of cardiovascular imaging for myocardial injury in hospitalized COVID-19 patients	EUR HEART J CARDIOVASC IMAGING	2020
38	Cui S.	Prevalence of venous thromboembolism in patients with severe novel coronavirus pneumonia	J THROMB HAEMOST	2020
39	Danzi GB.	Acute pulmonary embolism and COVID-19 pneumonia/ a random association?	EUR HEART J	2020
40	Dargent A.	Lung ultrasound score to monitor COVID-19 pneumonia progression in patients with ARDS	PLOS ONE	2020
41	Davoodi L.	COVID-19 Presented With Deep Vein Thrombosis; An Unusual Presenting	J INVEST MED HIGH IMPACT CASE REP	2020
42	Demelo-Rodriguez P.	Incidence of asymptomatic deep vein thrombosis in patients with COVID-19 pneumonia and elevated D-dimer levels	THROMB RES	2020
43	Denault AY.	A proposed lung ultrasound and phenotypic algorithm for the care of COVID-19 patients with acute respiratory failure	CAN J ANESTH	2020
44	Deng Q.	Suspected myocardial injury in patients with COVID-19/ Evidence from front-line clinical observation in Wuhan, China	INTERN J CARDIOL	2020
45	Deng Q.	Semiquantitative lung ultrasound scores in the evaluation and follow-up of critically ill patients with COVID-19/ a single-center study	ACTA RADIOL	2020
46	Di Serafino M.	The lung ultrasound; facts or artifacts? In the era of COVID-19 outbreak	RADIOL MED	2020
47	Dong D.	The role of imaging in the detection and management of COVID-19; a review	IEEE XPLORE	2020
48	Drake DH.	Echocardiography in Pandemic; Front-Line Perspective, Expanding Role of Ultrasound, and Ethics of Resource Allocation	JASE	2020
49	Dubois-Silva A.	Pulmonary embolism and screening for concomitant proximal deep vein thrombosis in noncritically ill hospitalized patients with coronavirus disease 2019	INTERNAL EM MED	2020
50	Duclos G.	“No dose” lung ultrasound correlation with “low dose” CT scan for early diagnosis of SARS-COV-2 pneumonia	INTENSIVE CARE MED	2020
51	Dudea S.	Ultrasonography and SARS-CoV 2 infection; a review of what we know and do not yet know	MED ULTRASON	2020
52	Dugar S.	Spontaneous echo contrast in venous ultrasound of severe COVID-19 patients	INTENSIVE CARE MED	2020
53	Duggan NM.	BEST PRACTICE RECOMMENDATIONS FOR POINT-OF-CARE LUNG ULTRASOUND IN PATIENTS WITH SUSPECTED COVID-19	ULTRASOUND EM MED	2020
54	Duggan NM.	Using Lung Point-of-care Ultrasound in Suspected COVID-19; Case Series and Proposed Triage Algorithm	CLIN PRACT CASES EM MED	2020
55	Dweck MR.	Global evaluation of echocardiography in patients with COVID-19	EUR HEART J CARDIOVASC IMAGING	2020
56	Farrow II R.	Early Multi-organ Point-of-Care Ultrasound Evaluation of Respiratory Distress During SARS-CoV-2 Outbreak; Case Report	CLIN PRACT CASES EM MED	2020
57	Fiala MJ.	Ultrasound in COVID-19; a timeline of ultrasound findings in relation to CT	CLIN RADIOL	2020
58	Flower L.	The Use of Point-of-Care Lung Ultrasound and Echocardiography in the Management of Coronavirus Disease 2019 (COVID-19)	J CARDIOTHORAC VASC ANESTH	2020
59	Fox S.	Point-of-care ultrasound and COVID-19	CLEV CLIN MED J	2020
60	Gackowski A.	Echocardiography during the coronavirus disease 2019 (COVID-19) pandemic; expert opinion of the Working Group on Echocardiography of the Polish Cardiac Society	KARDIOLOGIA POLSKA	2020
61	Gargani L.	Why, when, and how to use lung ultrasound during the COVID-19 pandemic/ enthusiasm and caution	EUR HEART J CV IMAGING	2020
62	GIannini A.	Lung ultrasound for pregnant women admitted to ICU for Covid-19 pneumonia	MINERVA ANEST	2020
63	Goldsmith AJ.	Remodeling Point-of-care Ultrasound Education in the Era of COVID-19	AEM EDU AND TRAINING	2020
64	Gravell RJ.	Radiological manifestations of COVID-19; key points for the physician	BR J HOSP MED	2020
65	Guarracino F.	Lung, Heart, Vascular, and Diaphragm Ultrasound Examination of COVID-19 Patients; A Comprehensive Approach	J CARDIOTHOR VASC ANESTH	2020
66	AIUM	Guidelines for Cleaning and Preparing External- and Internal-Use Ultrasound Transducers and Equipment Between Patients as well as Safe Handling and Use of Ultrasound Coupling Gel	AIUM	2020
67	Haaksma ME.	LUS findings in patients with novel SARS-CoV2	medXRiv	2020
68	Hankins A.	Point of care lung ultrasound is useful when screening for CoVid-19 in Emergency Department patients	medXRiv	2020
69	Hasanin A.	Evaluation of fluid responsiveness during COVID-19 pandemic/ what are the remaining choices?	J ANESTH	2020
70	Heldeweg M.	Lung ultrasound and computed tomography to monitor COVID-19 pneumonia in critically ill patients/ a two-center prospective cohort study	RES SQUARE preprint	2020
71	Helms J.	High risk of thrombosis in patients in severe SARS-CoV-2 infection; a multicenter prospective cohort study	INTENSIVE CARE MED	2020
72	Hu H.	Coronavirus fulminant myocarditis treated with glucocorticoid and human immunoglobulin	EUR HEART J	2020
73	Hua A.	Life-threatening cardiac tamponade complicating myo-pericarditis in COVID-19	EUR HEART J	2020
74	Huang J.	Development of a COVID-19 Point-of-Care Ultrasound Protocol	JASE	2020
75	Huang Y.	A preliminary study on the ultrasonic manifestations of peripulmonary lesions of non-critical novel coronavirus pneumonia (COVID-19)	medXRiv	2020
76	Inchingolo R.	The diagnosis of pneumonia in a pregnant woman with coronavirus disease 2019 using maternal lung ultrasound	AM J OBSTETRICS	2020
77	Inciardi RM.	Cardiac Involvement in a Patient With Coronavirus Disease 2019 (COVID-19)	JAMA CARDIOL	2020
78	Jain SS.	Indications and Findings on Transthoracic Echocardiography in COVID-19	JASE	2020
79	Johri AM.	ASE Statement on PoCUS in COVID-19	JASE	2020
80	Jung EM.	Contrast enhanced ultrasonography (CEUS) to detect abdominal microcirculatory disorders in severe cases of COVID-19 infection; First experience	CLIN HEMORHEOL MICROCIRC	2020
81	Kalafat E.	Lung ultrasound and computed tomographic findings in pregnant woman with COVID-19	ULTRASOUND OBSTET GYNECOL	2020
82	Kaminski A.	Answering to the Call of Critically Ill Patients/ Limiting Sonographer Exposure to COVID-19 with Focused Protocols	JASE	2020
83	Kariyanna PT.	Apical Takotsubo Cardiomyopathy in a COVID-19 Patient Presenting with Stroke; A Case Report and Pathophysiologic Insights	AM J MED CASE REPORTS	2020
84	Kariyanna PT.	Thrombus in Transit and Impending Pulmonary Embolism Detected on POCUS in a Patient with COVID-19 Pneumonia	AM J MED CASE REPORTS	2020
85	Khalili N.	Lung Ultrasound in COVID-19 Pneumonia; Prospects and Limitations	ACADEMIC RADIOLOGY	2020
86	Kiamanesh O.	Lung Ultrasound for Cardiologists in the Time of COVID-19	CAN J CARDIOL	2020
87	Kim DJ.	Just_the_facts_recommendations_on_pointofcare_ultrasound_use_and_machine_infection_control_during_the_coronavirus_disease_2019_pandemic	CJEM	2020
88	Kirkpatrick AW.	Lung ultrasonography in a woman with COVID-19/ This examination could be remote	CMAJ	2020
89	Klok FA.	Incidence of thrombotic complications in critically ill ICU patients with COVID-19	THROMB RES	2020
90	Koleialat I.	Clinical characteristics of acute lower extremity deep venous thrombosis diagnosed by duplex in patients hospitalized for coronavirus disease 2019	J VASC SURG VEN LYMPH DISORDERS	2020
91	Koratala A.	Need for Objective Assessment of Volume Status in Critically Ill Patients with COVID-19; The Tri-POCUS Approach	CARDIORENAL MED	2020
92	Kraemer AXE.	POCUS series; point-of-care lung ultrasound in patients with COVID-19	NETH J CRIT CARE	2020
93	Kulkarni S.	Point-of-care lung ultrasound in intensive careduring the COVID-19 pandemic	CLIN RADIOL	2020
94	Kumar A.	Lung Ultrasound Findings in Patients Hospitalized with Covid-19	medXRiv	2020
95	Lazzeri C.	Pulmonary embolism and screening for concomitant proximal deep vein thrombosis in noncritically ill hospitalized patients with coronavirus disease 2019	INTERNAL EM MED	2020
96	Lepri G.	The emerging role of lung ultrasound in COVID-19 pneumonia	EUR J RHEUMATOLOGY	2020
97	Li J.	Response to letter to the editor/ “Lung Ultrasound early detection and monitoring in Covid-19 Pneumonia; fact and fiction”	QJM	2020
98	LI J.	Serial bedside lung ultrasonography in a critically ill COVID-19 patient	QJM	2020
99	Liu R.	Ultrasound on the Frontlines of COVID-19/ Report From an International Webinar	ACAD EMERG MED	2020
100	Liu X.	Critical Care Ultrasonography and Its Application for COVID-19	ADV ULTRASOUND DIAG THER	2020
101	Llitjos JF.	High incidence of venous thromboembolic events in anticoagulated severe COVID-19 patients	J THROMB HAEMOST	2020
102	Lodigiani C.	Venous and arterial thromboembolic complications in COVID-19 patients T admitted to an academic hospital in Milan, Italy	THROMB RES 2020	2020
103	Lomoro P.	COVID-19 pneumonia manifestations at the admission on chest ultrasound, radiographs, and CT; single-center study and comprehensive radiologic literature review	EUR J RADIOL OPEN	2020
104	Longchamp A.	Venous thromboembolism in critically Ill patients with COVID-19/ Results of a screening study for deep vein thrombosis	RES PRACT THROMB HAEMOST	2020
105	Lu W.	A Clinical Study of Noninvasive Assessment of Lung Lesions in Patients with Coronavirus Disease-19 (COVID-19) by Bedside Ultrasound	ULTRASCHALL MED	2020
106	Lu W.	Response to Letter to the editor/ Lung Ultrasound in COVID-19 Patients – More Shadows Than Information – Letter to the Editor on the Article “W. LU et al. Ultraschall in Med. 2020 Apr 15	ULTRASCHALL MED	2020
107	Ma IWY.	Canadian Internal Medicine Ultrasound (CIMUS) Recommendations Regarding Internal Medicine Point-of-Care Ultrasound (POCUS) use during Coronavirus (COVID-19) pandemic	CAN J GEN INTERN MED	2020
108	Ma J.	Critical Care Ultrasound_ A Necessary and Mandatory Tool for Novel Coro-navirus Pneumonia	ADVANCED ULTRASOUND DIAG THER	2020
109	Manivel V.	CLUE/ COVID-19 Lung Ultrasound in Emergency Department	EMERG MED AUSTRALAS	2020
110	Mathews BK.	Clinical Progress Note/ Point-of-Care Ultrasound Applications in COVID-19	J HOSP MED	2020
111	McDermott C.	Combatting COVID-19; Is Ultrasound an Important Piece in the Diagnostic Puzzle?	RESEARCHGATE Preprint	2020
112	McMahon SR.	Tablet-Based Limited Echocardiography to Reduce Sonographer Scan and Decontamination Time during the COVID-19 Pandemic	JASE	2020
113	Mendel J.	Current Concepts Imaging in COVID-19 and the Challenges for Low and Middle Income Countries	J GLOBAL RADIOL	2020
114	Middeldorp S.	Incidence of venous thromboembolism in hospitalized patients with COVID-19	J THROMB HAEMOSTH	2020
115	McMahon SR.	Tablet-Based Limited Echocardiography to Reduce Sonographer Scan and Decontamination Time during the COVID-19 Pandemic	JASE	2020
116	Mendel J.	Current Concepts Imaging in COVID-19 and the Challenges for Low and Middle Income Countries	J GLOBAL RADIOL	2020
117	Michard F.	Haemodynamic Monitoring and Management in COVID-19 Intensive Care Patients/ An International Survey	ANESTH CRIT CARE MED	2020

PoCUS4COVID

Expedited Delphi consensus process on the use of
Point of Care UltraSound for COVID Diagnosis and Management

118	Middeldorp S.	Incidence of venous thromboembolism in hospitalized patients with COVID-19 - J THROMB HAEMOSTH 2020.pdf	J THROMB HAEMOSTH	2020
119	Mojoli F.	Our recommendations for acute management of COVID-19	CRIT CARE	2020
120	Moller-Sorensen H.	COVID-19 Assessment with Bedside Lung Ultrasound in a Population of Intensive Care Patients Treated with Mechanical Ventilation and ECMO	DIAGNOSTICS	2020
121	Mongodi S.	LUNG ULTRASOUND IN PATIENTS WITH ACUTE RESPIRATORY FAILURE REDUCES CONVENTIONAL IMAGING AND HEALTH CARE PROVIDER EXPOSURE TO COVID-19	ULTRASOUND MED BIOL	2020
122	Moore S.	Point of care and intensive care lung ultrasound; A reference guide for practitioners during COVID-19	RADIOGRAPHY	2020
123	Moores LK.	Prevention, Diagnosis, and Treatment of VTE in Patients With Coronavirus Disease 2019 CHEST Guideline and Expert Panel Report	CHEST	2020
124	Moro F.	How to perform lung ultrasound in pregnant women with suspected COVID-19 - ULTRASOUND OBSTET GYNECOL 2020.pdf	ULTRASOUND OBSTET GYNECOL	2020
125	Mouhand FHM.	Frequency of Abnormalities Detected by Point-of-Care Lung Ultrasound in Symptomatic COVID-19 Patients/ Systematic Review and Meta-Analysis	AM J TROP MED HYG	2020
126	Nahum J.	Venous Thrombosis Among Critically Ill Patients With Coronavirus Disease 2019 (COVID-19)	JAMA NETWORK OPEN	2020
127	Nauka PC.	Deep venous thrombosis in a non-critically ill patient with novel COVID-19 infection	THROMB RES	2020
128	Nicoara A.	Specific Considerations for the Protection ofPatients and Echocardiography ServiceProviders When Performing Perioperative orPeriprocedural TransesophagealEchocardiography during the 2019 NovelCoronaviru	JASE	2020
129	Nouvenne A.	Lung Ultrasound in COVID-19 Pneumonia; Correlations with Chest CT on Hospital admission	RESPIRATION	2020
130	Obi At.	Practical diagnosis and treatment of suspected venous thromboembolism during COVID-19 Pandemic	J VASC SURG VEN LYMPH DIS	2020
131	Omar YA.	Routine use of Point-of-Care lung ultrasound during the COVID-19 pandemic	MED INTENSIVA	2020
132	Osterwalder J.	COVID-19 – More Lung Pocus and Sparing Use of Stethoscope, Chest X-Ray and Lung CT	PRAXIS	2020
133	Pacileo M.	The role of echocardiography in SARS-CoV-2 pandemic; a compromise among appropriateness, safety and clinical impact	MONALDI ARCH CHEST DIS	2020
134	Palmese F.	Daily Evaluation of COVID-19 Patients Primarily Based on Lung Ultrasound/ In Times of Emergency, It's Time to Change Some Paradigms	AM J TROP MED HYG	2020
135	Para JR.	Point-of-care Lung Ultrasound Is More Sensitive than Chest Radiograph for Evaluation of COVID-19	WEST J EM MED	2020
136	Pascarella	The evolution of ultrasound role in COVID-19 pandemic/ from triage to screening	ANESTH ANALG	2020
137	Pata D.	Chest Computed Tomography and Lung Ultrasound Findings in COVID-19 Pneumonia; A Pocket Review for Non-radiologists	FRONT MED	2020
138	Peh WM.	Lung ultrasound in a Singapore COVID-19 intensive care unit patient and a review of its potential clinical utility in pandemic	J ULTRASOUND	2020
139	Peyrony O.	Accuracy of Emergency Department Clinical Findings for Diagnosis of Coronavirus Disease 2019	ANN EM MED	2020
140	Picard MH.	Echocardiography in the Time of COVID-19	JASE	2020
141	Pierce CW.	Clarifying the role of lung ultrasonography in COVID-19 respiratory disease	CMAJ	2020
142	Piliego C.	The Ultrasound-Guided Triage/ A New Tool for Prehospital Management of COVID-19 Pandemic	ANESTH ANALG	2020
143	Piscaglia F.	Benefits, Open questions and Challenges of the use of Ultrasound in the COVID-19 pandemic era. The views of a panel of worldwide international experts	ULTRASCHALL MED	2020
144	Pivetta E.	Lung ultrasound for the diagnosis of SARS-CoV-2 pneumonia in the Emergency Department	ANN EMERG MED	2020
145	Poggiali E.	Can Lung US Help Critical Care Clinicians in the Early Diagnosis of Novel Coronavirus (COVID-19) Pneumonia?	RADIOLOGY	2020
146	Poggiali E.	Triage decision-making at the time of COVID-19 infection/ the Piacenza strategy	INTERN EM MED	2020
147	Qian F.	A valuable and affordable handheld ultrasound in combating COVID-19	CRIT CARE	2020
148	Qian-Yi P.	Findings of lung ultrasonography of novel corona virus pneumonia during the 2019–2020 epidemic	INTENSIVE CARE MED	2020
149	Qian-Yi P.	Using echocardiography to guide the treatment of novel coronavirus pneumonia	CRIT CARE	2020
150	Rajkumar R.	Point of Care Ultrasound During the Covid-19 Pandemic Should Screen for Inter-Atrial Shunt	ADVANCED ULTRASOUND DIAGN THER	2020
151	Reisinger N.	Lung ultrasound/ a valuable tool for the assessment of dialysis patients with COVID-19	CLIN EXPERIM NEPHROL	2020
152	Ren B.	Extremely High Incidence of Lower Extremity Deep Venous Thrombosis in 48 Patients With Severe COVID-19 in Wuhan	CIRCULATION	2020
153	Roth KR.	Computed tomography and point-of-care ultrasound findings in 2 COVID-19 patients	J AM COLL EM PHYS OPEN	2020
154	Roy S.	Deep Learning for Classification and Localization of COVID-19 Markers in Point-of-Care Lung Ultrasound	IEEE TRANSACT MED IMAGING 2020	2020
155	Rubulotta F.	Technologies to Optimize the Care of Severe Coronavirus Disease 2019 Patients for Health Care Providers Challenged by Limited Resources	ANESTH ANALG	2020
156	Santiago M.	Use of US to expedite diagnosis of PE in COVID-19 Patient	VISUAL J EM MED	2020
157	Santoliquido A.	Incidence of Deep Vein Thrombosis among non-ICU Patients Hospitalized for COVID-19 Despite Pharmacological Thromboprophylaxis	J THROMB HAEMOSTH	2020
158	Schultz MJ.	Challenges and Opportunities for Lung Ultrasound in Novel Coronavirus Disease (COVID-19)	AM J TROP MED HYG	2020
159	Scopettuolo G.	Vascular access in COVID-19 patients; Smart decisions for maximal safety	J VASC ULTRASOUND	2020
160	Shaw JA.	Lung Ultrasound in COVID-19; Not Novel, but Necessary	RESPIRATION	2020
161	Shokoohi H.	Lung ultrasound monitoring in patients with COVID-19 on home isolation	AM J EM MED	2020
162	Sikachi R.	Whole body point-care ultrasound for COVID-19/ a multi-system approach to a multi-system disease	ANAESTHESIA	2020
163	Smallwood N.	Should point-of-care ultrasound become part of healthcare worker testing for COVID?	ROYAL COLLEGE PHYS	2020
164	Smargiassi A.	Lung ultrasonography for early management of patients with respiratory symptoms during COVID-19 pandemic	J ULTRASOUND	2020
165	Smith MJ.	Point-of-care lung ultrasound in patients with COVID-19 – a narrative review	ANAESTHESIA	2020
166	Soccorsa S.	Thoracic ultrasound and SARS-COVID-19; a pictorial essay	J ULTRASOUND	2020
167	Sohail S.	Rational and practical use of imaging in COVID-19 pneumonia	PAK J MED SCI 2020	2020
168	Soldati G.	Contrast-Enhanced Ultrasound in Patients With COVID-19 Pneumonia, Acute Respiratory Distress Syndrome, or Something Else?	J ULTRASOUND MED	2020
169	Soldati G.	On Lung Ultrasound Patterns Specificity in the Management of COVID-19 Patients	J ULTRASOUND MED	2020
170	Soldati G.	Proposal for international standardization of the use of lung ultrasound for COVID-19 patients; a simple, quantitative, reproducible method	J ULTRASOUND MED	2020
171	Sollazzo V.	Echocardiographic Features in Patients with Critical COVID-19 Requiring Endotracheal Intubation	INTERN J CLIN SKILLS	2020
172	Sperandeo M.	Diagnosis of coronavirus disease 2019 pneumonia in pregnant women/ can we rely on lung ultrasound?	AM J OBST	2020
173	Sperandeo M.	Lung Ultrasound early detection and monitoring in Covid-19 Pneumonia/ fact and fiction.	QJM	2020
174	Sperandeo M.	Lung Ultrasound in COVID-19 Patients – More Shadows Than Information – Letter to the Editor on the Article "W. LU et al. Ultraschall in Med. 2020 Apr 15	ULTRASCHALL MED	2020
175	Sultan LR.	A REVIEW OF EARLY EXPERIENCE IN LUNG ULTRASOUND IN THE DIAGNOSIS AND MANAGEMENT OF COVID-19	ULTRASOUND MED BIOL	2020
176	Szabo AI.	Pathophysiological background and clinical practice of lung ultrasound in COVID-19 patients: A short review	ANATOL J CARDIOL	2020
177	Szekely Y.	The Spectrum of Cardiac Manifestations in Coronavirus Disease 2019 (COVID-19) – a Systematic Echocardiographic Study	CIRCULATION	2020
178	Tan G.	USE OF LUNG ULTRASOUND TO DIFFERENTIATE CORONAVIRUS DISEASE 2019 (COVID-19) PNEUMONIA FROM COMMUNITY-ACQUIRED PNEUMONIA	ULTRASOUND MED BIOL	2020
179	Tavazzi G.	Myocardial localization of coronavirus in COVID-19 cardiogenic shock	EUR J HEART FAIL	2020
180	Tavazzi G.	Thrombotic events in SARS-CoV-2 patients; an urgent call for ultrasound screening	INTENSIVE CARE MED	2020
181	Tee A.	Contrast-enhanced ultrasound (CEUS) of the lung reveals multiple areas of microthrombi in a COVID-19 patient	INTENSIVE CARE MED	2020
182	Thalappillil R.	POCUS to Guide Fluid Therapy in COVID-19	J CARDIOTHORAC VASC ANESTH	2020
183	Thomas A.	Lung ultrasound findings in a 64-year-old woman with COVID-19	CMAJ	2020
184	Trauer MM.	Utility of Lung Ultrasound in COVID-19; A Systematic Scoping Review	medXRiv	2020
185	Trimaille A.	Venous thromboembolism in non-critically ill patients with COVID-19 infection	THROMB RES	2020
186	Trovato GM.	Usefulness of lung ultrasound imaging in COVID-19 pneumonia/ The persisting need of safety and evidences	ECHOCARDIOGRAPHY	2020
187	Tung Chen Y.	Acute pericarditis due to COVID-19 infection; An underdiagnosed disease?	MED CLIN	2020
188	Tung Chen Y.	CORRELATION BETWEEN CHEST COMPUTED TOMOGRAPHY AND LUNG ULTRASONOGRAPHY IN PATIENTS WITH CORONAVIRUS DISEASE 2019 (COVID-19)	medXRiv	2020
189	Tung Chen Y.	Lung ultrasound in the monitoring of COVID-19 infection	CLIN MED	2020
190	Tung-Chen Y.	THE ROLE OF POINT-OF-CARE ULTRASONOGRAPHY IN THE INITIAL CHARACTERIZATION OF COVID-19 PATIENTS/ RESULTS FROM A PROSPECTIVE MULTICENTRIC STUDY	medXRiv	2020
191	Vetrugno L.	B lines in COVID-19/ "Unspecificity" is not "meaningless"	ECHOCARDIOGRAPHY	2020
192	Vetrugno L.	Lung Ultrasound and the COVID-19 "Pattern" Not All That Glitters Today Is Gold Tomorrow	J ULTRASOUND MED	2020
193	Vetrugno L.	Our Italian experience using lung ultrasound for identification, grading and serial follow-up of severity of lung involvement for management of patients with COVID-19	ECHOCARDIOGRAPHY	2020
194	Vieira ALS.	Role of point-of-care ultrasound during the COVID-19 pandemic; our recommendations in the management of dialytic patients	ULTRASOUND J	2020
195	Volpicelli G.	Sonographic signs and patterns of COVID-19 pneumonia	ULTRASOUND J	2020
196	Volpicelli G.	What's new in lung ultrasound during the COVID-19 pandemic	INTENSIVE CARE MED	2020
197	Walsh P.	PoCUS is useful when screening for CoVID-19 in ED patients	medXRiv	2020
198	Wan DY.	Current practice and potential strategy in diagnosing COVID-19	EUR REV MED PHARMACOL SCI	2020
199	Wang E.	Chinese Association of Anesthesiologists Expert Consensus on the Use of Perioperative Ultrasound in Coronavirus Disease 2019 Patients	J CARDIOTHOR VASC ANESTH	2020
200	Ward RP.	Utilization and Appropriateness of Transthoracic Echocardiography in Response to the COVID-19 Pandemic	JASE	2020
201	Wong A.	Ultrasound in Times of COVID-19	HEALTHCARE MANAGEMENT	2020
202	Xing C.	Lung ultrasound findings in patients with COVID-19 pneumonia	CRIT CARE	2020
203	Yang Y.	Lung ultrasonography versus chest CT in COVID-19 pneumonia; a two-centered retrospective comparison study from China	INTENSIVE CARE MED	2020
204	Yassa M.	Lung Ultrasound Can Influence the Clinical Treatment of Pregnant Women With COVID-19	J ULTRASOUND MED	2020
205	Yasukawa K.	Point-of-Care Lung Ultrasound Findings in Patients with COVID-19 Pneumonia	AM J TROP MED	2020
206	Ye R.	Feasibility of a 5G-based robot-assisted remote ultrasound system for cardiopulmonary assessment of COVID-19 patients	CHEST	2020
207	Yusuf GT.	The use of contrast-enhanced ultrasound in COVID-19 lung imaging	J ULTRSOUND	2020
208	Youssef A.	Lung ultrasound in the coronavirus disease 2019 pandemic/ a practical guide for obstetricians and gynecologists	AM J OBST GYN	2020
209	Zeng JH.	First case of COVID-19 complicated with fulminant myocarditis; a case report and insights	INFECTION	2020
210	Zhang L.	Deep Vein Thrombosis in Hospitalized Patients With COVID-19 in Wuhan, China	CIRCULATION	2020
211	Zhang Li.	Bedside Focused Cardiac Ultrasound in COVID-19 from the Wuhan Epicenter; The Role of Cardiac Point-of-Care Ultrasound, Limited Transthoracic Echocardiography, and Critical Care Echocardiography	JASE	2020
212	Zieleskiewicz L.	Comparative study of lung ultrasound and chest computed tomography scan in the assessment of severity of confirmed COVID-19 pneumonia	INTENSIVE CARE MED	2020
213	Zieleskiewicz L.	Ultrasound findings in patients with COVID-19 pneumonia in early and late stages; Two case-report	ANESTH CRIT CARE PAIN MED	2020
214	Zotzmann V.	Are subpleural consolidations indicators for segmental pulmonary embolism in COVID-19?	INTENSIVE CARE MED	2020
