# Supplementary material for: Multi-organ point-of-care ultrasound for COVID-19 (PoCUS4COVID): international expert consensus
Source: Crit Care. 2020 Dec 24;24:702. doi: 10.1186/s13054-020-03369-5 (PMC7759024; doi:10.1186/s13054-020-03369-5)
Supplement: Supplementary file 2 — Additional file 1B. Consensus Methodology [file 13054_2020_3369_MOESM2_ESM.pdf]

Rand Rules for Voting

Introduction to RAND Appropriateness Method (RAM)

In collaboration with clinicians at the University of California at Los Angeles (UCLA), RAND Heath staff developed the RAND/UCLA Appropriateness Method to synthesize the scientific literature (evidence) and expert opinion on health care topics. This method has become a leading paradigm for quality assessment in medicine. It is also a mechanism for reaching formal agreement about how science should be interpreted in the real world. It makes it possible to set rules for determining best practices-guidelines that, when implemented, increase the value of health care management. The method was adopted by the European Commission BIOMED Concerted Action on the appropriateness of medical and surgical procedures and received wide acceptance as a reproducible, validated consensus development method in several countries. The basic concept of RAM is to have structured method in obtaining the panel decisions regarding ranking or regarding agreement on the appropriateness. The method incorporates modified Delphi technique that is carried out in a minimum 2 face-to-face rounds. This achieves the benefits of the interactions between the experts while keeping the benefits of the anonymity through the controlled feedback in the 2-rounds anonymous voting. The method establishes the panel judgment based on a reproducible statistical analysis of the panel's voting results, not only to establish agreement/disagreement but also to sensitively measure the degree of the agreement if present. For those who are specifically interested in gettin into depth of RAND methodology, a full manual can be found at http://www.rand.org/pubs/monograph_reports/MR1269

References:

1. http://www.rand.org/pubs/monograph_reports/MR1269 Last access 4th of february 2015
2. http://www.rand.org/health/surveys_tools/appropriateness.html. Last access 4th of february 2015
3. Fitch K, Bernstein SJ, Aguilar MD, Burnand B, LaCalle JR, Lazaro P, van het Loo M, McDonnell J, Vader JP, Kahan JP, (2001) The RAND/UCLA Appropriateness Method User's Manual. RAND Corporation, Arlington, VA, USA
4. Gonzalez N, Quintana JM, Lacalle JR, Chic S, Maroto D, (2009) Review of the utilization of the RAND appropriateness method in the biomedical literature (1999-2004). Gac Sanit 23: 232-237
5. Manesh R. Patel, John A. Spertus, Ralph G. Brindis et al. ACCF Proposed Method for Evaluating the Appropriateness of Cardiovascular Imaging. JACC Vol 46, Issue 8, 18 October 2005, Pages 1606–1613. doi:10.1016/j.jacc.2005.08.030

Panel voting following Rand appropriateness method using 9-points Likert Scale

- **Scale** 1-9
  - 9 =extremely appropriate
  - 1= extremely inappropriate
- **With 3 regions/zones:**
  - Inappropriate region: 1-3
  - Uncertain region: 4-6
  - Appropriate region: 7-9
- **The Likert Scale is used for voting on:**
  1. Judgment about outcome importance (9=critical 1=unimportant)
  2. Judgment about the transforming factors EtR (Evidence-to-Recommendation) or EtD (evidence-to-Decision) table. See EtR table please.
  3. Judgment about the overall appropriateness of draft recommendation (statement)
- **From analysis of voting** results the following is determined
  1. Presence of disagreement/agreement
  2. Degree of consensus
  3. Direction of recommendation (with or against)
  4. Strength of recommendation (weak or strong or No recommendation)
- **Disagreement** is defined by more than 30% of panelists have voted outside the 3 point region containing the median.
- **The degree of consensus** is driven from 3 factors
  1. Presence or absence of disagreement
  2. The median score
  3. The degree of dispersion of voters around the median (IQR and Integer needed to achieve majority percentage)

Term	Definition
**Perfect consensus**	All respondents agree on one number between 7-9
**Very good consensus**	Median and middle 50% (interquartile range) of respondents are found at one integer (*e.g.*, median and interquartile range are both at 8) or 80% of respondents are within one integer of the median (*e.g.*, median is 8, 80% respondents are from 7 to 9)
**Good consensus**	50% of respondents are within one integer of the median (*e.g.*, median is 8, 50% of respondents are from 7 to 9) or 80% of the respondents are within two integers of the median (*e.g.*, median is 7, 80% of respondents are from 5 to 9).
**Some consensus**	50% or respondents are within two integers of the median (*e.g.*, median is 7, 50% of respondents are from 5 to 9) or 80% of respondents are within three integers of the median (*e.g.*, median is 6, 80% of respondents are from 3 to 9).
**No consensus**	All other responses. Any median with disagreement

Recommendation Strength & Direction

Strong recommendation

Definition: has to have all of 3 conditions:

1. No disagreement (voters are ≥70%) AND
2. the Degree of consensus is at least **very good** (voters with ≥80% at 1 integer) AND
3. median score is NOT in the undetermined middle zone (median is NOT in 4-6 zone so it is either in the zone 7-9 or zone 1-3)

2 classes of strong recommendations:

- "**Strong with**" if median score is =7-9
- "**Strong against**" if median score is = 1-3

The word **recommend** will be used for strong recommendation

The word **must, should or to** depends on the degree of consensus (as shown in the table below)

Conditional/Weak recommendation

Definition: 3 conditions

1. No disagreement (voters are ≥70%) AND
2. The Degree of consensus is "**good or some consensus**" with any median score OR median score is 4-6 with any degree of consensus. AND
3. median score is NOT in the undetermined middle zone (median is NOT in 4-6 zone so it is either in the zone 7-9 or zone 1-3)

- "**Weak against**" if middle 50% Interquartile range = 1 - ≤3
- "**Weak with**" if middle 50% Interquartile range = 4-9

No recommendation

Definition: either of 3 conditions

1. Disagreement (voters are ≥70%) OR
2. No consensus OR
3. Median in the middle region (4-6) with any degree of consensus.

This table summarize the relation between the degree of consensus, the strength of recommendations and the wording to be used

Degree of consensus	Strength of recommendation	Wording [Function of voting]
Perfect consensus	Strong	recommend – must/to be/will
Very good consensus	Strong	recommend – should be/can
Good consensus	Weak/Conditional	suggest – to do
Some consensus	Weak/Conditional	suggest - may do
No consensus Disagreement	NO	No recommendation was made regarding

Figure 1. Algorithm for applying RAND rules on 2-rounds of voting for panel decisions

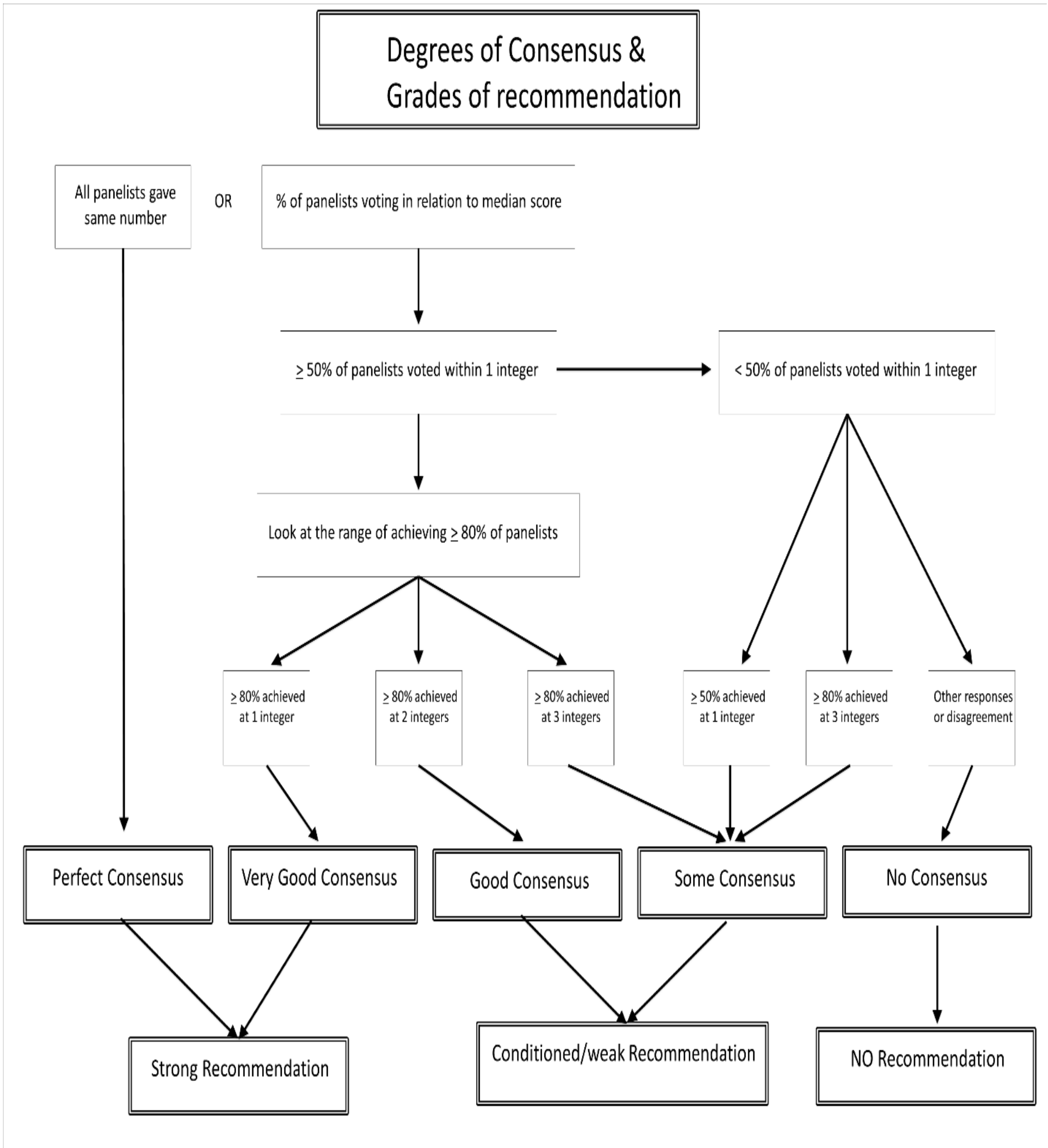

Examples:

• Disagreement: if > 30% of panelists voted outside the zone of the median.

• Examples of consensus degree & recommendation strength

- 50% of respondents are within one integer of the median
    - (e.g., median is 8, 50% of respondents are from 7 to 9)
- 50% or respondents are within two integers of the median
    - (e.g., median is 7, 50% of respondents are from 5 to 9)
- 80% of respondents are within three integers of the median
    - (e.g., median is 6, 80% of respondents are from 3 to 9

EtR (Evidence-to-Recommendation) Table

Round # **Domain code** **Statement (draft recommendation) code**

1. Does the statement address strategy that has clinical outcome? If No then GO DIRCTLY to appropriateness (approval) voting sheet (separate sheet).

2. If YES, then does the statement address a strategy that has more than one outcome?

☐ YES ☐ NO[†]

The 5 Transforming Factors	Voting	Explanation
**1. Problem Priority /Importance**<br>Outcome 1________its rank is<br>Outcome 2________ its rank is<br>Outcome 3________ its rank is<br>List here the previously determined rank of outcome importance and See SoF tables.<br>The more critical is the outcome or the highest the priority of problem, the more likely is a strong recommendation[$].	**Rank of priority/ most important outcome**<br>☐ 9<br>☐ 8<br>☐ 7<br>☐ 6<br>☐ 5<br>☐ 4<br>☐ 3<br>☐ 2<br>☐ 1	**Your notes:**
**2. Level of Quality of Evidence (LQE)** [$]<br>Outcome 1___its evidence quality__<br>Outcome 2___its evidence quality__<br>Outcome 3___its evidence quality__<br>See SoF tables. The higher the quality of evidence, the more likely is a strong recommendation.	**The overall quality across outcomes**<br>☐ High<br>☐ moderate<br>☐ low	If multiple outcomes, overall quality will be based on that of the most important outcome (e.g. of the critical). If multiple equal outcomes (e.g. all have equal importance), then it will have the least estimated quality.<br>**Your notes:**

**3. Benefit /Harm balance**<br>The larger the difference between the desirable and undesirable consequences and the certainty around that difference, the more likely a strong recommendation. The smaller the net benefit and the lower the certainty for that benefit, the more likely is a conditional/weak recommendation.	☐ 9<br>☐ 8<br>☐ 7<br>☐ 6<br>☐ 5<br>☐ 4<br>☐ 3<br>☐ 2<br>☐ 1	9 =extremely favorable balance<br>1=extremely unfavorable balance with 3 regions 7-9 favorable, 4-6 uncertain and 1-3 unfavorable<br>**<u>Your notes/concerns:</u>**
**4. Benefit/Burden balance**<br>The higher the resource consumed of an intervention or burden related to the decision, the more likely is a conditional/weak recommendation. Are the resources consumed/burden worth the expected benefit?	☐ 9<br>☐ 8<br>☐ 7<br>☐ 6<br>☐ 5<br>☐ 4<br>☐ 3<br>☐ 2<br>☐ 1	9 =extremely favorable balance<br>1=extremely unfavorable balance with 3 regions 7-9 favorable, 4-6 uncertain and 1-3 unfavorable,<br>**<u>Your notes/concerns:</u>**
**5. Certainty/Concerns about PEAF ***<br>**<u>P</u>references/<u>E</u>quity/<u>A</u>cceptability/ <u>F</u>easibility**<br>The greater the certainty around these 4 variables, the more likely is a strong recommendation. The more concerns, the likely is weak/conditional recommendation	☐ 9<br>☐ 8<br>☐ 7<br>☐ 6<br>☐ 5<br>☐ 4<br>☐ 3<br>☐ 2<br>☐ 1	9 =extremely certain about PEAF<br>1=extremely concerned about PEAF with 3 regions 7-9 certain, 4-6 uncertain and 1-3 concerned<br>**<u>Your notes/concerns:</u>**

Also called **EtD** (Evidence-to-Decision) table. *†* If No (i.e., one outcome) then please list in column 2 the previously determined level of quality of evidence (LQE) that is presented in Summary of Findings (SoF) table. $ Level of Quality of Evidence (LQE) also called CEE=Confidence in the Estimate of Effect ***** Certainty around preferences means expecting average patient will choose this action and minimal variability are expected in patient choices. Concerns means expected wide variability in patient preferences towards this recommendation. Similarly, certainty related to equity (patient accessibility to care), acceptability (by various stakeholders) and feasibility (infrastructure). The more likely the certainty, the more likely is a strong recommendation. The more concerns about any PEAF factors, the likely is weak/conditional recommendation.

○
