# Supplementary material for: Multi-organ point-of-care ultrasound for COVID-19 (PoCUS4COVID): international expert consensus
Source: Crit Care. 2020 Dec 24;24:702. doi: 10.1186/s13054-020-03369-5 (PMC7759024; doi:10.1186/s13054-020-03369-5)
Supplement: Supplementary file 3 — Additional file 1C. Consensus Results and Summary Recommendations [file 13054_2020_3369_MOESM3_ESM.pdf]

PoCUS4COVID

Expedited Delphi consensus process on the use of
Point of Care UltraSound for COVID Diagnosis and Management

PICO ELEMENTS

1. **POPULATIONS**
    a. MILDLY-ILL COVID-19 PATIENTS
    b. COVID-19 PATIENTS with RESP FAILURE
    c. COVID-19 PATIENTS with SEVERE EXTRA-PULM SIGNS & SYMPTOMS
2. **INTERVENTIONS**
    a. LUS ALONE
    b. MULTI-ORGAN POCUS
3. **COMPARATORS**
    a. NO LUNG IMAGING
    b. CXR
    c. CT LUNG
4. **OUTCOMES**
    a. MORE EFFECTIVE DX SARS-COV2 INFECTION
    b. MORE EFFECTIVE TRIAGE/DISPOSITION
    c. MORE ACCURATE PULMONARY DX
    d. MORE ACCURATE CVS DX
    e. MORE ACCURATE VTE DX
    f. MORE EFFECTIVE USE OF MECH VENT
    g. MORE APPROPRIATE FLUID MANAGEMENT
    h. MORE EFFECTIVE MONITORING
    i. BETTER INFECTION CONTROL

Each PICO statement requires you to express your agreement with it based on a Likert scale (0-9): 0-3 = disagreement, 4-6 = uncertainty, 7-9 agreement.

The wording of the PICO questions during the 2 rounds of the this streamlined Delphi Technique was not changed substantially.

1. POPULATIONS

a. **MILDLY-ILL COVID-19 PATIENTS*** - presenting to Emergency Department with fever, myalgia, cough, sore throat or other mild symptoms, including exertional dyspnea but not clinically evident acute respiratory failure/hypoxemia, shock, or any other life-threatening or potentially life-threatening condition)

b. **COVID-19* PATIENTS* with RESPIRATORY FAILURE -** including both "Silent Hypoxia" & clinically-overt respiratory failure, either at presentation or along disease course

c. **COVID-19* PATIENTS *with SEVERE EXTRA-PULMONARY SIGNS & SYMPTOMS -** such as shock, altered level of consciousness, stroke, suspected acute thromboembolic disease, acute kidney failure, acute peripheral ischemic disease, either at presentation or along disease course

* **COVID-19 patients** refers to patients to be screened for SARS-CoV2 infection at first encounter, or already diagnosed with SARS-CoV2 infection . This is where the PICO Outcomes choices apply: e.g. with the Outcome "More effective diagnosis" and "More effective triage/Disposition" (Outcomes a, b) "COVID-19 PATIENTS" refers to patient at first encounter. However, "More effective monitoring" and all the others Outcomes (Outcomes c-i.), may apply either to first-encounter/unknown SARS-CoV2 infection, or already microbiologically demonstrated SARS-CoV2 infection).

2. INTERVENTIONS

a. **LUS ALONE** – Clinically-integrated lung ultrasound and no other PoCUS applications

b. **MULTI-ORGAN PoCUS** - Clinically-integrated multi-modal, multi-system point-of-care ultrasound, including:

- Lung Ultrasound
- Focused Cardiac Ultrasound (+/- hemodynamic measurements)
- Abdominal scanning of great vessels and kidneys, bladder, gallbladder, liver (potentially including Doppler sampling of kidney artery/vein, Hepatic veins and Portal vein for diagnosis of systemic venous congestion), FAST exam
- Extremity Duplex compression evaluation for DVT

3. COMPARATORS

a. **NO LUNG IMAGING** – Standard/Institutional diagnostic/monitoring clinical workup without the use of any imaging modalities

b. **CXR** – Standard/Institutional diagnostic/monitoring clinical workup including Chest X-Ray

c. **CT LUNG -** Standard/Institutional diagnostic/monitoring clinical workup including Chest CT-Scan, plus angio-CT scan if indicated (thoracic great vessels, PE)

4. OUTCOMES

a. MORE EFFECTIVE DIAGNOSIS OF SARS-COV2 INFECTION

- Higher sensitivity, specificity, & accuracy in diagnosing SARS-CoV2 infection

b. MORE EFFECTIVE TRIAGE/DISPOSITION – May include:

- Higher accuracy at assessment of potential SARS-CoV2 disease
- Higher accuracy in severity assessment of COVID-19 patients
- Potentially resulting in more rapid provision and more appropriate level of care and cohorting of patients

c. MORE ACCURATE PULMONARY DIAGNOSIS – May include:

- Better assessment of pulmonary manifestations and progression of disease
- Better assessment of pulmonary conditions other than COVID-19 pneumonia
- Better assessment of superimposed lung conditions

d. MORE ACCURATE CVS DIAGNOSIS – May include:

- Better assessment of cardiovascular manifestations (shock, cardiac arrest, stroke, peripheral ischemia of the limbs or splanchnic circulation) and progression of disease
- Better assessment of cardiac complications

e. MORE ACCURATE VTE DIAGNOSIS

- Better assessment of venous thromboembolic manifestations (PE, DVT) & progression of disease

f. MORE EFFECTIVE USE OF MECHANICAL VENTILATION – May include:

- Better assessment of need for MV (non-invasive/invasive)
- More efficient use of MV (help in choice of ventilator modality/settings)
- Better choice of inadequacy of mechanical ventilation and need for extracorporeal support
- Potentially resulting in fewer complications of MV

g. MORE APPROPRIATE FLUID MANAGEMENT

- Better assessment of need of, responsiveness and tolerance to intravenous fluids

h. **MORE EFFECTIVE MONITORING –** May include:

- Better assessment of lung disease course
- Better assessment of lung aeration & recruitment
- Better assessment of hemodynamics
- Better assessment of cardiac disease course
- Better assessment of thromboembolic disease course
- Better assessment of weaning from mechanical ventilation
- Better monitoring of extracorporeal circulation

i. BETTER INFECTION CONTROL – May include:

- Reduced in-hospital viral spread
- Lower rate of infection amongst healthcare workers

PoCUS4COVID

Expedited Delphi consensus process on the use of
Point of Care UltraSound for COVID Diagnosis and Management

DELPHI ROUNDS RESULTS - SYNOPSIS

ROUND 1 - Synopsis

		Strength of Consensus				Direction of Agreement		
	N	VERY GOOD CONSENSUS	GOOD CONSENSUS	SOME CONSENSUS	NO CONSENSUS	AGREE	AMBIV	DISAGREE
Section 1	43	25,6%	49,2%	20,9%	7,0%	86,0%	6,9%	6,9%
Section 2	50	34,0%	46,0%	20,0%	0,0%	64,0%	10,0%	6,0%
Section 2	32	31,3%	59,4%	9,4%	0,0%	93,8%	0,0%	6,2%
Section 4	4	0,0%	100,0%	0,0%	0,0%	50,0%	50,0%	0,0%
TOTALS	129	30,7%	49,6%	17,3%	2,4%	86,6%	8,7%	4,7%

ROUND 2 - Synopsis

		Strength of Consensus				Direction of Agreement		
	N	VERY GOOD CONSENSUS	GOOD CONSENSUS	SOME CONSENSUS	NO CONSENSUS	AGREE	AMBIV	DISAGREE
Section 1	43	51,2%	25,6%	18,6%	7,0%	83,7%	9,3%	7,0%
Section 2	50	34,0%	54,0%	8,0%	4,0%	84,0%	12,0%	4,0%
Section 2	32	56,3%	31,3%	12,5%	0,0%	93,8%	6,3%	0,0%
Section 4	4	0,0%	75,0%	25,0%	0,0%	100,0%	0,0%	0,0%
TOTALS	129	44,2%	39,5%	13,2%	3,9%	86,8%	9,3%	3,9%

PoCUS4COVID

Expedited Delphi consensus process on the use of
Point of Care UltraSound for COVID Diagnosis and Management

PoCUS4COVID Domains

DOMAIN	PoCUS APPLICATION
1	DIAGNOSIS OF SARS-COV2 INFECTION
2	TRIAGE/DISPOSITION
3	DIAGNOSIS OF COVID-19 PNEUMONIA
4	CARDIOVASCULAR DIAGNOSIS
5	SCREENING AND DIAGNOSIS OF THROMBOEMBOLIC DISEASE
6	PoCUS AND RESPIRATORY SUPPORT STRATEGIES
7	MANAGEMENT OF FLUID ADMINISTRATION
8	MONITORING OF COVID-19 PATIENTS
9	INFECTION CONTROL, TECHNIQUES, TECHNOLOGY AND PROTOCOLS

PoCUS4COVID

Expedited Delphi consensus process on the use of
Point of Care UltraSound for COVID Diagnosis and Management

RESULTS and SUMMARY RECOMMENDATIONS

DOMAIN 1 - PoCUS for Diagnosis of SARS-CoV2 infection

1 - SARS DX	TOTAL		MILD	RESP FAIL	XTR-PULM
	N = 18	%	N = 7	N = 7	N = 4
VG	0	0,0%	0	0	0
G	10	55,6%	3	5	2
S	6	33,3%	3	1	2
NC	2	11,1%	1	1	0
AGREE	10	55,6%	4	4	3
AMBIV	2	11,1%	2	2	1
DISAGREE	5	27,8%	1	1	0

GRADEing		N = 11	IBID		CONS
2A	2B	3			
5	4	2	3		G

DOMAIN 2 - PoCUS as a Tool for Triage/Disposition

2 - TRG/DISP	TOTAL		MILD	RESP FAIL	XTR-PULM
	N = 18	%	N = 7	N = 7	N = 4
VG	5	27,8%	2	1	2
G	11	61,1%	4	5	2
S	2	11,1%	1	1	0
NC	0	0,0%	0	0	0
AGREE	16	88,9%	6	6	4
AMBIV	2	11,1%	1	1	0
DISAGREE	0	0,0%	0	0	0

GRADEing		N = 14			CONS
2A	2B	3			G
1	7	6			

DOMAIN 3 - PoCUS as a Tool for Triage/Disposition

3 - PULM DX	TOTAL		MILD	RESP FAIL	XTR-PULM
	N = 18	%	N = 7	N = 7	N = 4
VG	7	38,9%	4	2	1
G	4	22,2%	0	3	1
S	4	22,2%	1	1	2
NC	3	16,7%	2	1	0
AGREE	13	72,2%	5	5	3
AMBIV	2	11,1%	0	1	1
DISAGREE	3	16,7%	2	1	0

GRADEing		N = 53			CONS
2A	2B	3			G
5	18	28			

Recommendations

1) We suggest using PoCUS, and especially LUS (presence of heterogeneous B-line clusters, pleural line irregularities, subpleural consolidations), and appropriately integrate the information with clinical assessment to diagnose COVID-19 pneumonia **(LQE II-B, Very Good Agreement).**

2) When CT-scan is not accessible or appropriate, we suggest using LUS to aid the diagnosis of COVID-19 pneumonia in suspected cases **(LQE II-B, Good Agreement).**

3) In patients with high pre-test probability for COVID-19 and LUS findings suggestive of pneumonia, a negative nasal/oropharyngeal RT-CR may not be used to exclude COVID-19, and LUS findings, further raising suspicion, should prompt repeat testing with better yield **(LQE II-B, Good Agreement).**

4) We do *not* recommend using PoCUS and LUS alone to rule out SARS-CoV-2 infection in suspected COVID-19 (LQE II-B, Good Agreement).

5) After thorough examination of all lung fields and intercostal spaces a bilateral A-pattern suggests absence of pneumonia in suspected or confirmed SARS-CoV-2 infection **(LQE III, Good Agreement).**

6) We suggest multi-organ PoCUS integrated with other clinical information for triaging and risk stratification of suspected COVID-19 at initial presentation **(LQE II-B, Good Agreement).**

DOMAIN 4 - FoCUS, AND COMPREHENSIVE ECHOCARDIOGRAPHY FOR THE CARDIOVASCULAR DIAGNOSIS IN COVID-19 PATIENTS

4 - CVS DX	TOTAL		MILD	RESP FAIL	XTR-PULM
	N = 12	%	N = 4	N = 4	N = 4
VG	10	83,3%	4	3	3
G	2	16,7%	0	1	1
S	0	0,0%	0	0	0
NC	0	0,0%	0	0	0
AGREE	12	100,0%	4	4	4
AMBIV	0	0,0%	0	0	0
DISAGREE	0	0,0%	0	0	0

GRADEing		N = 26			CONS
2A	2B	3			
1	10	15	49		VG

Recommendations

7) We suggest FoCUS and/or echocardiography assessment in moderate-severe COVID-19 as it may change clinical management or provide information that could be lifesaving **(LQE II-B, Very Good Agreement).**

8) We suggest FoCUS and/or echocardiography for assessment of hemodynamic instability in moderate-severe COVID-19 **(LQE II-B, Very Good Agreement).**

9) We recommend FoCUS and echocardiography to diagnose RV and LV systolic dysfunction and cardiac tamponade as etiology of hemodynamic instability in COVID-19 **(LQE II-B, Very Good Agreement).**

10) We suggest using FoCUS/echocardiography to guide hemodynamic management in severe COVID-19 **(LQE II-B, Very Good Agreement).**

<u>DOMAIN 5</u> - PoCUS FOR SCREENING AND DIAGNOSIS OF COVID-19 PATIENTS FOR VENOUS THROMBOEMBOLIC DISEASE (VTE)

5 - VTE DX	TOTAL		MILD	RESP FAIL	XTR-PULM
	N = 12	%	N = 4	N = 4	N = 4
VG	9	75,0%	3	3	3
G	2	16,7%	1	1	1
S	1	8,3%	0	0	0
NC	0	0,0%	0	0	0
AGREE	12	100,0%	4	4	4
AMBIV	0	0,0%	0	0	0
DISAGREE	0	0,0%	0	0	0

GRADEing		N = 32			CONS
2A	2B	3	12		VG
10	12	10			

Recommendations

11) Because critically ill COVID-19 patients have high risk for VTE, we suggest regular screening for DVT, including central vessels with catheters, independent of oxygenation and coagulation **(LQE II-A, Very Good Agreement).**

12) In moderate-severe COVID-19 with hemodynamic worsening or sudden instability we suggest FoCUS for prompt investigation of acute cor-pulmonale **(LQE II-B, Very Good Agreement).**

13) In moderate-severe COVID-19 we suggest that echocardiographic indices of worsening RV function and/or increased pulmonary artery pressure may indicate PE **(LQE II-A, Very Good Agreement).**

DOMAIN 6 - PoCUS and respiratory support strategies [including mechanical Ventilation] in patients with COVID-19 Pneumonia

6 - RESP SUPP	TOTAL		MILD	RESP FAIL	XTR-PULM
	N = 11	%	N = 0	N = 7	N = 4
VG	4	36,4%	0	2	2
G	7	63,6%	0	5	2
S	0	0,0%	0	0	0
NC	0	0,0%	0	0	0
AGREE	10	90,9%	0	6	4
AMBIV	1	9,1%	0	1	0
DISAGREE	0	0,0%	0	0	0

GRADEing		N = 20			CONS
2A	2B	3	35		G
2	11	7	36		
			41		

Recommendations

14) We suggest multi-organ PoCUS including LUS over no imaging to guide respiratory support in COVID-19 with respiratory failure (i.e. ventilation, prone positioning, PEEP, recruitment maneuvers) **(LQE II-A, Good Agreement).**

15) In addition to standard respiratory monitoring, we suggest LUS over CXR and equally to CT, to guide clinical decisions on respiratory support in COVID-19 with respiratory failure **(LQE II-B, Good Agreement).**

16) We suggest multi-organ PoCUS over LUS alone for decisions about respiratory support in COVID-19 with respiratory failure **(LQE II-B, Good Agreement).**

DOMAIN 7 - POCUS FOR MANAGEMENT OF FLUID ADMINISTRATION IN COVID-19 PATIENTS

7 - FLD MAN	TOTAL		MILD	RESP FAIL	XTR-PULM
	N = 18	%	N = 7	N = 7	N = 4
VG	11	61,1%	4	3	4
G	5	27,8%	1	4	0
S	2	11,1%	2	0	0
NC	0	0,0%	0	0	0
AGREE	16	88,9%	6	6	4
AMBIV	2	11,1%	1	1	0
DISAGREE	0	0,0%	0	0	0

GRADEing		N = 9			CONS
2A	2B	3	5		VG
0	7	2			

Recommendations

17) We suggest FoCUS to screen for severe hypovolemia in moderate-severe COVID-19 at presentation, while Doppler-based fluid-responsiveness indices may be used for subsequent management **(LQE II-A, Very Good Agreement).**

18) We suggest that LUS alone is not sufficient as a screening tool for pulmonary congestion in moderate-severe COVID-19 **(LQE III, Very Good Agreement).**

19) We suggest that LUS alone is not sufficient to judge the appropriateness of fluid administration in moderate-severe COVID-19 **(LQE II-B, Very Good Agreement).**

20) In moderate-severe COVID-19 we suggest multi-organ PoCUS to monitor efficacy of fluid removal, by not only LUS findings of reduction of B-pattern areas, but also echocardiographic signs of resolution of volume overload and decreasing LV filling pressures **(LQE II-B, Very Good Agreement).**

DOMAIN 8 - PoCUS AS MONITORING TOOL FOR PATIENTS WITH COVID-19

8 - MONITOR	TOTAL		MILD	RESP FAIL	XTR-PULM
	N = 18	%	N = 7	N = 7	N = 4
VG	11	61,1%	5	3	3
G	7	38,9%	2	4	1
S	0	0,0%	0	0	0
NC	0	0,0%	0	0	0
AGREE	18	100,0%	7	7	4
AMBIV	0	0,0%	0	0	0
DISAGREE	0	0,0%	0	0	0

GRADEing		N = 49			CONS
2A	2B	3	10		VG
4	29	16	11		
			25		
			49		
			51		
			64		
			66		
			67		

Recommendations

21) We suggest serial LUS for respiratory monitoring in moderate-severe COVID-19 **(LQE II-B, Very Good Agreement).**

22) We suggest multi-organ PoCUS integrated with other clinical and biochemical variables, in preference to CXR for investigation of respiratory deterioration in moderate-severe COVID-19. **(LQE II-A, Very Good Agreement).**

23) We suggest multi-organ PoCUS over LUS alone to detect respiratory deterioration and guide treatment in moderate-severe COVID-19. **(LQE II-B, Very Good Agreement).**

24) We suggest a prompt assessment of clinical deterioration with LUS for a timely and accurate bedside diagnosis of pneumothorax in severe COVID-19 **(LQE II-B, Very Good Agreement).**

25) We suggest LUS for early identification of ventilator-associated pneumonia in severe COVID-19 **(LQE II-B, Very Good Agreement).**

26) We suggest multi-organ PoCUS over CXR and CT to assess readiness for weaning, predict success, and diagnose the cause(s) of weaning failure in COVID-19 **(LQE II-B, Very Good Agreement).**

27) We suggest FoCUS, and/or echocardiography for hemodynamic monitoring in moderate-severe COVID-19 **(LQE II-A, Very Good Agreement).**

28) We suggest integrating PoCUS-derived information with data from other devices used for hemodynamic monitoring in severe COVID-19 **(LQE II-B, Very Good Agreement).**

29) We suggest PoCUS assessment for pre-renal causes of AKI, including hemodynamics and venous congestion in COVID-19 **(LQE II-B, Very Good Agreement).**

PoCUS4COVID

Expedited Delphi consensus process on the use of
Point of Care UltraSound for COVID Diagnosis and Management

DOMAIN 9 - PoCUS and Infection Control, PoCUS technique, technology, protocols

9 - INF CON	N = 4	%
VG	0	0,0%
G	3	75,0%
S	1	25,0%
NC	0	0,0%
AGREE	4	100,0%
AMBIV	0	0,0%
DISAGREE	0	0,0%

GRADEing			N = 23	CONS
2A	2B	3		G
4	14	5		

Recommendations

30) We suggest using laptop/tablet/pocket-sized devices with adequate imaging capabilities that are easier to protect from viral contamination in COVID-19 **(LQE II-B, Good Agreement).**

31) For diagnostic accuracy, quality control and obtaining second opinions, we suggest performance of standardized PoCUS examinations in COVID-19 **(LQE II-B, Good Agreement).**

32) We recommend reporting PoCUS studies and recording, storage and archiving of diagnostic images and cine-clips **(LQE II-B, Good Agreement).**

33) We suggest using tele-ultrasound for remote guidance and consultations in COVID-19. Simple audio-visual communication devices (e.g. smartphones) can facilitate this **(LQE II-B, Good Agreement).**

34) We suggest POCUS over CXR and CT, where appropriate, to reduce environmental spread of infection and risk of infection to healthcare workers in COVID-19 **(LQE II-A, Good Agreement).**

35) We recommend strict adherence to manufacturers' guidance for cleaning and disinfection of equipment used for COVID-19 **(LQE II-A, Good Agreement).**

36) We suggest brief and targeted ultrasound examinations to minimize cross-infection in COVID-19 **(LQE II-B, Good Agreement).**
