# Supplementary material for: Multi-organ point-of-care ultrasound for COVID-19 (PoCUS4COVID): international expert consensus
Source: Crit Care. 2020 Dec 24;24:702. doi: 10.1186/s13054-020-03369-5 (PMC7759024; doi:10.1186/s13054-020-03369-5)
Supplement: Supplementary file 9 — Additional file 7. PoCUS-empowered triage in respiratory failure during COVID-19 Pandemic. Conceptual framework of point-of-care ultrasound (PoCUS) use for the triage of dyspneic and/or hypoxemic patients, during the SARS-CoV-2 pandemic: the diagram does not represent an algorithm but rather a framework for potentially developing protocols according to local/institutional clinical practices, policies and regulations. It does not either provide a list of conclusive diagnosis or specific treatments, but suggests how to integrate at best PoCUS in the workflow of this specific setting [file 13054_2020_3369_MOESM9_ESM.pdf]

Additional File 7 – PoCUS EMPOWERED TRIAGE IN RESPIRATORY FAILURE during COVID-19 PANDEMIC

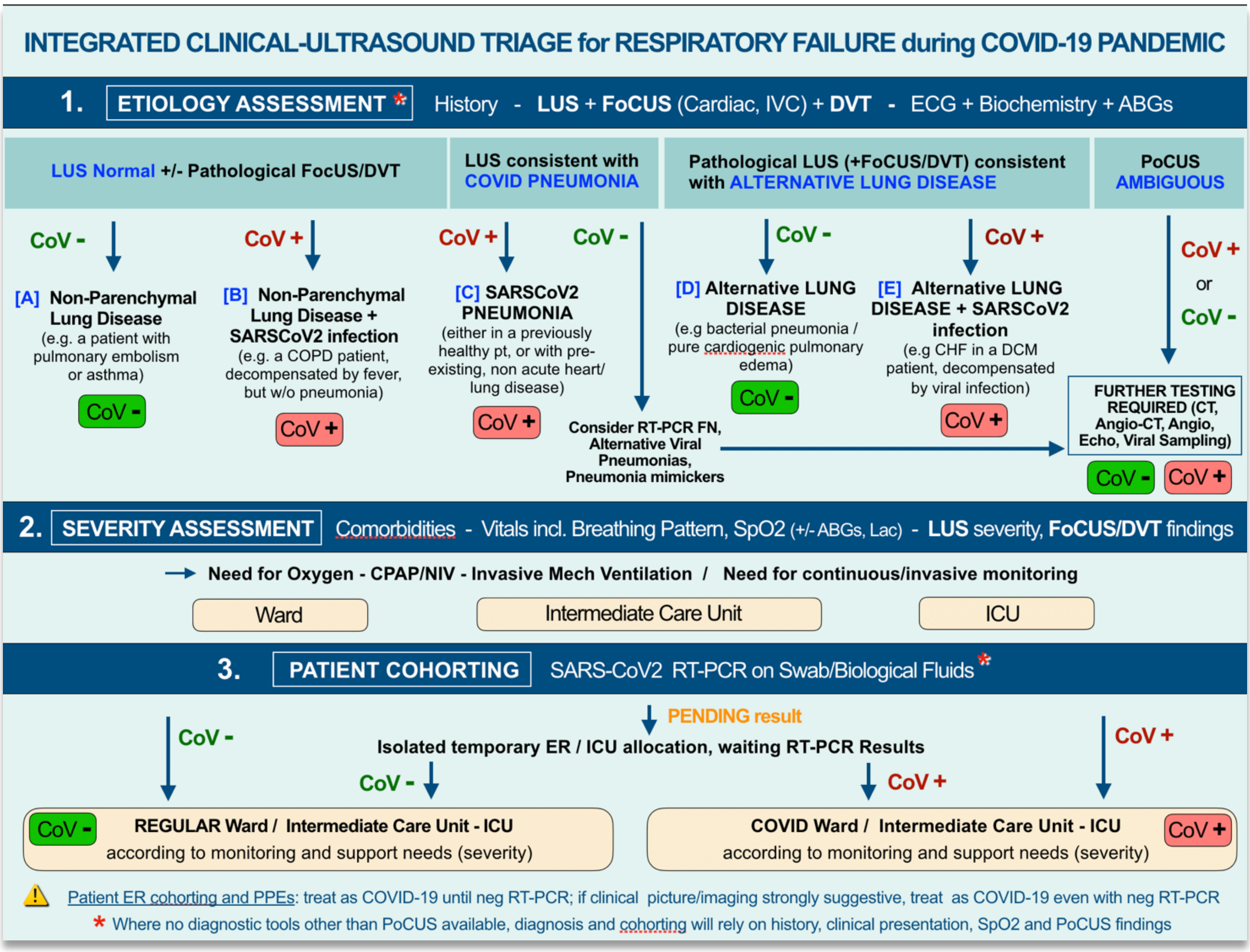

Conceptual framework of Point of Care Ultrasound (PoCUS) use for the triage of dyspneic and/or hypoxemic patients, during the SARS-CoV2 pandemic. The diagram does not represent an algorithm but rather a framework for potentially developing protocols according to local/institutional clinical practices, policies and regulations. It does not either provide a list of conclusive diagnosis or specific treatments, but rather provides some examples of diagnoses while suggesting how to integrate at best PoCUS in the workflow of this specific setting.

There are 3 COMPONENTS OF AN APPROPRIATE TRIAGE in this scenario, that require simultaneous assessment and consistent decisions for a correct patient treatment and allocation:

- **THE DIAGNOSIS** - the specific cause (or at least initially the pathophysiology) of respiratory failure is investigated. The goal is to reach the correct diagnosis as soon as possible and initiate appropriate treatment on likely etiology.
- **THE SEVERITY OF PATIENT CONDITION** - the degree of respiratory compromise, including the potential for rapid deterioration, is assessed. The goal is twofold: to set up immediate treatment for vital support, where required, according to ACLS priorities; to identify the specific need in terms of level of monitoring and intensity of care, and in the end admit the patient to the most appropriate facility (ER observation area, ER ward, pulmonology or cardiology ward, intermediate care unit/high-dependency unit, intensive care unit).

- **THE PRESENCE/ABSENCE OF SARS-CoV2 INFECTION** – in a situation of worldwide ongoing viral pandemic and especially in phases of local significant prevalence of the disease, every patient with respiratory failure and a more-than-low pre-test probability for SARS-CoV2 infection must be screened for it, when microbiological testing available. Without referring here to the diagnostic accuracy of specific tests (RT-PCR on nasal/oral swabs, on respiratory secretions, or Specific Antibodies blood sampling), identification of infected patients is paramount to avoid infection spreading among patients and healthcare workers, and to reach the specific diagnosis of COVID-19 pneumonia, or of COVID-19 (i.e. otherwise symptomatic SARS-CoV2 infection) contributing to the respiratory failure.

The **CONTRIBUTION OF POCUS IN ASSESSING THESE COMPONENTS** can be considered, as follows:

1. **ETIOLOGY ASSESSMENT** - The combined use of Lung Ultrasound (LUS), Focused Cardiac Ultrasound (FoCUS, including IVC evaluation) and of compressive ultrasound of the deep veins (DVT) has shown high accuracy in the diagnosis of respiratory failure causes, outperforming standard approaches without ultrasound. It can thus greatly aid the differential diagnosis of COVID-19 pneumonia and other causes of respiratory failure. In terms of PoCUS applied according to ABCDE priorities, LUS will generally be performed first, and its findings will orientate patient categorization with respect to the lung condition as:
    - *Normal* (overall LUS normal pattern, i.e. bilateral A-pattern detected upon a thorough LUS exam, “lawnmower”-style, at all intercostal spaces of each quadrant, including parascapular dorsal areas)
    - *COVID-Pneumonia* (suggestive LUS pattern of SARS-CoV2 pneumonia; pending definitive RT-PCR results for confirming the infection)
    - *Alternative lung disease* (suggestive LUS pattern of cardiac cause or parenchymal lung disease other than SARS-CoV2 pneumonia)
    - *Ambiguous* (LUS pathological, but showing ambiguous diagnostic pattern or mixed/overlapping patterns)

    Pending definitive results for SARS-CoV2 RT-PCR, integration of these LUS patterns with FoCUS (+/- DVT) findings, with the clinical picture, with ECG and biochemistry (including blood cell count, Procalcitonin, C-Reactive Protein, coagulation panel, D-Dimer, liver and kidney function tests, and cardiac biomarkers, when appropriate) can lead to the final diagnosis:
    [A] – *Non-parenchymal lung disease*
    [B] – *Non-parenchymal lung disease, but with concurrent SARS-CoV2 infection*
    [C] – *COVID-19 pneumonia as cause of respiratory failure*
    [D] – *Parenchymal lung disease alternative to COVID-19 pneumonia*
    [E] – *Parenchymal lung disease alternative to COVID-19 pneumonia but with concurrent SARS-CoV2 infection/pneumonia*
    Some examples of the diagnoses associated to these categories are reported in parentheses in the diagram.

    [B] o [E] are conditions that will present as respiratory failure, either with febrile COVID-19 decompensating a pre-existing respiratory/cardiac disease, or with mild COVID-19 pneumonia as concurrent but not principal respiratory failure cause.

    Where a LUS pattern suggestive of viral pneumonia is found, but at first screening SARS-CoV2 RT-PCR on swab is negative, the possibilities include a false negative RT-PCR test, an alternative viral cause of pneumonia, or a less common diffuse interstitial lung disease (potentially mimicking COVID-19 pneumonia in this pandemic context, also considering potential limited access to past medical history) (e.g. connective tissue disorders, hypersensitivity pneumonias, alveolar proteinosis, lung cancer, etc.).

Ambiguous PoCUS findings (either due overlapping disease or an insufficiently informative exam, e.g. low echogenicity / deep lung lesions not abutting the surface), or any uncertainty along the diagnostic process, will trigger further testing. Lung CT-scan and Echocardiography become then first-line tests, with potential high diagnostic yield.

In scarce-resource settings, ONLY where availability of diagnostic tests other than PoCUS is limited, microbiological testing is not available, and the pre-test probability for COVID-19 is high, the diagnosis may rely on medical history, clinical presentation, SpO2 and PoCUS findings. The high-prevalence of the disease in the pandemic likely provides this approach with sufficient positive predictive value, although specific evidence is awaited.

2. **SEVERITY ASSESSMENT**- LUS signs of diffuse and/or relevant lung disease (especially but not only in COVID-19 pneumonia) or FoCUS detection of significant underpinning cardiac disease can provide key information on the severity of patient conditions, or on a potential rapid worsening, thus contributing to correct choice of patient monitoring and intensity of care. Additionally, consider that the detection of deep venous thrombosis at presentation may be a sign of severity, and an indication to early anticoagulation therapy, instead of simple thromboprophylaxis.

3. **PATIENT COHORTING** - The contribution of PoCUS to patient cohorting is obvioulsy less relevant: LUS findings with 100% positive predictive value for COVID-19 pneumonia are yet to be validated, and SARS-CoV2 infection without pneumonia can only be diagnosed through microbiological testing. Where false negative RT-PCR test is an issue, a strong clinical-sonographic suspicion for COVID-19 pneumonia should still prompt patient isolation and PPE use, and consideration of the patient as SARS-CoV2 infected, until otherwise proven. This regardless of multiple negative SARS-CoV2 swab tests.

LUS = lung ultrasound; FoCUS = Focused Cardiac UltraSound; DVT = compressive ultrasound for deep venous thrombosis; PoCUS = Point of Care UltraSound; ECG = Electrocardiogram; CoV+ = SARS-CoV2 positive RT-PCR test; CoV- = SARS-CoV2 positive RT-PCR test; COPD = chronic obstructive pulmonary disease; CHF = congestive heart failure; DCM = dilated cardiomyopathy; RT-PCR = reverse transcriptase polymerase chain reaction assay; FN = false negative; CT = computed tomography; Angio-CT = angiorgraphic computed tomography; Angio = Angiography, coronary; Echo = Echocardiography; SpO2 = pulsioximetric saturation; ABG = arterial blood gas analysis; Lac = arterial blood lactatemia; CPAP = Continuous Positive Airway Pressure; NIV = NonInvasive Ventilation; ER = emergency room; ICU = intensive care unit; PPE = personal protective equipment.
